# Integrated ^13^C Metabolic Flux and Multi-Omics Analyses Reveal Central Carbon and Redox Constraints in a Salt-Sensitive *Chromochloris zofingiensis* Mutant

**DOI:** 10.3390/md24100337

**Published:** 2026-09-27

**Authors:** Zhixiong Li, Yuanyuan Ren, Xiaozhen Huang, Shufang Yang, Jia Wang, Lei Qin, Xue Lu, Han Sun

**Affiliations:** 1 Institute for Advanced Study, Shenzhen University, Shenzhen 518071, China; gdldlzx@163.com (Z.L.); ysf_nbj@163.com (S.Y.); 2 School of Life Sciences, Nanchang University, Nanchang 330031, China; 3 Shenzhen Foreign Languages School, Shenzhen 518000, China; 4 School of Resources and Environment, Nanchang University, Nanchang 330031, China; 5 Discipline of Chinese and Western Integrative Medicine, Jiangxi University of Chinese Medicine, Nanchang 330004, China; 6 College of Food Science and Engineering, Ocean University of China, Qingdao 266003, China; 7 Institute of New Materials and Advanced Manufacturing, Beijing Academy of Science and Technology, Beijing 100089, China; luxue@bjast.ac.cn

**Keywords:** *Chromochloris zofingiensis*, cGMP-dependent protein kinase, salinity acclimation, ^13^C metabolic flux analysis, metabolomics, transcriptomics, redox homeostasis

## Abstract

Saline cultivation can reduce freshwater demand and reshape the production physiology of high-value microalgae, yet the metabolic basis of strain-specific salinity tolerance remains unclear. Here, the astaxanthin-producing green microalga *Chromochloris zofingiensis* was examined by comparing the wild type (*Cz*-WT) with a cGMP-dependent protein kinase (PKG)-deficient mutant (*Cz-pkg*) under 100 mM NaCl. Physiological measurements were integrated with parallel ^13^C-glucose tracing, ^13^C metabolic flux analysis, LC–MS metabolomics, and time-series RNA sequencing. Relative to *Cz-pkg*, *Cz*-WT showed higher normalized fluxes from phosphoenolpyruvate to pyruvate and oxaloacetate, greater pyruvate-to-acetyl-CoA conversion, and enhanced tricarboxylic acid (TCA)-cycle activity, suggesting a greater potential to support energy and reducing-equivalent metabolism. *Cz-pkg* exhibited lower glucose-6-phosphate and 6-phosphogluconate levels, early NADH depletion, and reduced pools of several amino-acid- and nucleotide-related metabolites. At 24 h, 141 genes were co-upregulated and 228 co-downregulated in both strains, involving photosynthetic electron transport, antioxidant metabolism, glycolysis/gluconeogenesis, amino-acid metabolism, and terpenoid biosynthesis. Phosphatidylglycerophosphate synthase was upregulated in both strains, supporting membrane-lipid remodeling. Overall, the data provide flux-level evidence that the salt-sensitive mutant is constrained in glycolysis–TCA cycle coupling and energy–redox homeostasis during salinity acclimation. Direct PKG substrates and causal phosphorylation events were not examined in this study.

## 1. Introduction

Microalgae are essential primary phototrophic producers in ecosystems and contribute significantly to global net primary production (NPP) [1]. Compared with terrestrial plants, microalgae can fix CO_2_ using sunlight with efficiencies up to an order of magnitude higher, making them critical for global carbon capture and climate regulation. Through photosynthesis, microalgae convert light energy into chemical energy for growth and assimolate inorganic carbon, primarily CO_2_ and bicarbonate (HCO_3_^−^), into cellular biomass, thereby contributing to biological carbon sequestration [2,3]. Recent developments in microalgae-based semiartificial photosynthesis further highlight the potential of microalgae as adaptable platforms for solar-driven carbon fixation and sustainable biomanufacturing [4].

Microalgal growth and metabolite accumulation are highly dependent on the environmental conditions. Factors such as nutrient limitation [5], high light intensity [6], and temperature fluctuations [7] can significantly affect the physiological metabolic state of microalgae. Salinity is a crucial environmental factor that substantially influences the physiology of microalgae. Excessively high or low salt concentrations can alter cellular osmotic pressure, induce the accumulation of reactive oxygen species (ROS), disrupt intracellular carbon flow, and profoundly affect cell growth, metabolism, and composition [8]. Given the increasing scarcity of freshwater resources, elucidating the mechanisms by which microalgae respond to salinity acclimation has significant theoretical and practical value for cultivating microalgae in high-salinity environments or seawater.

Different microalgae show markedly different tolerances to salt concentration, and salinity can have a dual effect on the same strain. Moderate salinity level may stimulate growth and metabolism, whereas excessive salt conditions can impair photosynthesis, disturb redox homeostasis, and inhibit growth [9]. Recent omics-based studies have further revealed extensive metabolic and regulatory reprogramming during salinity acclimation. Study in the soil green alga *Desmochloris* sp. FACHB-3271 showed that salinity treatment induced time-dependent physiological and transcriptional responses involving photosynthesis, energy metabolism, osmotic regulation, and signaling pathways [10]. Integrated transcriptomic and metabolomic analyses in *Chlorella* sp. DT025 showed substantial redistribution of carbon among carbohydrate, amino acid, nucleotide, and lipid metabolism under NaCl treatment [11], while physiological and transcriptomic analyses of *Chlamydomonas* sp. demonstrated that acclimation to contrasting salinities varied with growth stage [12]. Moreover, transcriptomic, phosphoproteomic, and functional genomic analyses in *Chlamydomonas reinhardtii* identified multiple cellular compartments and signaling processes involved in osmotic-stress acclimation [13]. More recently, comprehensive multi-omics analysis of *Scenedesmus obliquus* revealed coordinated changes in photosynthesis, central metabolism, lipid remodeling, and redox regulation under salinity stress [14]. These studies highlight the value of integrating multiple molecular layers to resolve microalgal salinity responses.

*Chromochloris zofingiensis* (*C. zofingiensis*) is a unicellular haploid green alga reported from temperate terrestrial habitats and exhibits considerable metabolic and trophic flexibility [15,16]. It can grow under autotrophic, heterotrophic, and mixotrophic conditions and is amenable to high-density cultivation and the production of high-value metabolites [17,18]. In heterotrophic fed-batch cultures with glucose as the carbon source, the biomass can reach up to 53 g/L [19]. In addition to its capacity for high biomass accumulation, *C. zofingiensis* can accumulate high-value metabolites, such as lipids, astaxanthin, and other carotenoids [20], demonstrating significant potential for both research and application. Previous studies have shown that wild-type *C. zofingiensis* (*Cz*-WT) exhibits high survival under high salinity conditions and accumulates abundant astaxanthin and triacylglycerol [21]. Preliminary research identified a mutant named *Cz-pkg*, produced by chemical mutagenesis of the *Cz*-WT using the mutagen *N*-methyl-*N*’-nitro-*N*-nitrosoguanidine (MNNG) [22]. This mutant retains its photosynthetic system active in the presence of glucose and shows significant differences from the wild type in pigment and lipid metabolism. Evidence suggests that inactivated cGMP-dependent protein kinase (PKG) in this mutant may be involved in plant stress response pathways [23]. More recently, Ren et al. reported that PKG inactivation compromises salt tolerance in *C. zofingiensis* and is associated with altered metabolic adaptation, antioxidant responses, lipid biosynthesis, and astaxanthin accumulation under salinity treatment [24]. However, the metabolic-flux basis underlying this differential salinity response remains insufficiently resolved.

Despite recent advances in multi-omics analyses, flux-level understanding of carbon redistribution during microalgal salinity acclimation remains limited. Recent ^13^C-fluxomics studies have demonstrated the ability of isotope-based approaches to resolve changes in intracellular carbon allocation that cannot be inferred from metabolite abundance alone. For example, Deshpande et al. used ^13^C-fluxomics to identify substantial redistribution of carbon through the phosphoenolpyruvate (PEP)/oxaloacetate (OAA) node and toward carbohydrate and lipid synthesis in *Scenedesmus obliquus* [25]. Here, we integrated growth analysis, ^13^C metabolic flux analysis (^13^C-MFA), metabolite profiling, and time-series transcriptomics to compare central-carbon acclimation in *Cz*-WT and *Cz-pkg* under 100 mM NaCl treatment, focusing on strain-associated differences in glycolysis–tricarboxylic acid cycle (TCA cycle, also known as the citric acid cycle or Krebs cycle) coupling, redox metabolism, and carbon allocation during salinity acclimation.

## 2. Results and Discussion

### 2.1. Differential Growth Responses to Salinity and Selection of 100 mM NaCl

Salt stress causes osmotic imbalance and oxidative stress in microalgal cells, thereby affecting growth and metabolic activity [9]. Previous studies have shown that *C. zofingiensis* possesses some salt tolerance and can accumulate lipids and astaxanthin under moderate salt treatment [21]. To systematically evaluate the effect of salinity on growth and photosynthesis, we treated *Cz*-WT and *Cz-pkg* with a range of NaCl concentrations (0–200 mM) under mixotrophic conditions (5 g/L glucose).

As shown in Figure 1a, biomass accumulation in *Cz*-WT was slightly stimulated at low-to-moderate salt concentrations (0–100 mM NaCl). After 4 days of salt treatment, the control (0 mM) cultures reached a biomass of 2.67 ± 0.08 g/L. In contrast, the 100 mM NaCl-treated group reached 3.17 ± 0.09 g/L, slightly above the control. These results suggest that moderate salt concentrations may enhance osmotic regulation and promote metabolic redistribution, thereby promoting *Cz*-WT biomass accumulation. However, at 200 mM NaCl, the *Cz*-WT biomass dropped to 2.05 ± 0.07 g/L, indicating significant biomass accumulation inhibition at high salinity. This result is consistent with our previous findings [21].

In contrast, *Cz-pkg* showed stronger growth inhibition as salinity increased, reaching only 0.55 ± 0.08 g/L after 4 d at 200 mM NaCl (Figure 1b), about 25% of the control and not significantly different from the initial inoculum, indicating virtually arrested growth. Such dose-dependent salinity responses are also observed in other green algae. In *Chlamydomonas reinhardtii*, 100 mM NaCl has been used as a nonlethal osmotic challenge that elicits extensive transcriptional and cellular acclimation, whereas stronger salt treatments can markedly inhibit growth and photosynthetic performance [13,26]. These observations emphasize that the physiological outcome of salinity depends strongly on stress intensity and cellular acclimation capacity. Based on the differential but nonlethal response of the two strains, 100 mM NaCl was selected for the subsequent ^13^C-MFA and multi-omics analyses.

### 2.2. ^13^C-MFA Reveals Distinct Central Carbon Flux Allocation Under 100 mM NaCl Treatment

Based on the biomass responses to different salt concentrations, we selected 100 mM NaCl as the standard salinity acclimation condition for detailed analysis of metabolic flux changes under mixotrophic culture. Using ^13^C-MFA, we quantitatively analyzed the flux distributions of key carbon metabolic pathways and the production of primary metabolites in both strains under salinity acclimation. We constructed a metabolic flux model by measuring biomass composition of *Cz*-WT and *Cz-pkg* under 100 mM NaCl treatment, including fatty acid (Tables S1 and S2), amino acid (Table 1), and nucleic acid (DNA and RNA, Table 2) components. The nucleotide composition was inferred from the *C. zofingiensis* genome, which has a G + C content of 51% [15]. Based on these data, simplified biomass equations were formulated to provide key input parameters for flux calculations (Equations (1) and (2)).

For each strain under 100 mM NaCl treatment, amino acid isotopomer data obtained from the ^13^C-labeled glucose experiments were used as isotopic constraints in Metran to estimate intracellular metabolic fluxes. The biomass equations used for flux calculation were as follows:

(1) *Cz*-WT: Biomass + 9.456435 ADP = 0.048163 rCTP + 0.048163 rGTP + 0.046274 rATP + 0.046274 rUTP + 9.456435 ATP + 0.017208 dGTP + 0.017208 dCTP + 0.016533 dATP + 0.016533 dTTP + 0.101050 C16:0 + 0.001959 C16:1 + 0.022286 C16:2 + 0.026013 C16:3 + 0.004888 C16:4 + 0.035396 C18:0 + 0.216751 C18:1 + 0.102618 C18:2 + 0.005362 C18:3 + 0.005010 C18:4 + 2.133642 ADPG + 0.198932 Asp + 0.000171 Asn + 0.098648 Thr + 0.068038 Ser + 0.000135 Gln + 0.302788 Glu + 0.087376 Gly + 0.147332 Ala + 0.018163 Cys + 0.150484 Val + 0.032110 Met + 0.098493 Ile + 0.038098 Tyr + 0.166049 Leu + 0.102813 Phe + 0.133723 Lys + 0.022332 His + 0.000120 Trp + 0.171152 Arg + 0.080189 Pro + 0.521332 Glyc3P + 0.018018 DXP.

(2) *Cz-pkg*: Biomass + 10.26344 ADP = 0.049535 rCTP + 0.049535 rGTP + 0.047592 rATP + 0.047592 rUTP + 10.26344 ATP + 0.017076 dGTP + 0.017076 dCTP + 0.016406 dATP + 0.016406 dTTP + 0.098807 C16:0 + 0.003374 C16:1 + 0.022653 C16:2 + 0.022171 C16:3 + 0.009640 C16:4 + 0.026509 C18:0 + 0.136402 C18:1 + 0.077117 C18:2 + 0.075672 C18:3 + 0.005302 C18:4 + 2.086752 ADPG + 0.198168 Asp + 0.000174 Asn + 0.104282 Thr + 0.094992 Ser + 0.000139 Gln + 0.300146 Glu + 0.124881 Gly + 0.158182 Ala + 0.010238 Cys + 0.119612 Val + 0.036243 Met + 0.081100 Ile + 0.061360 Tyr + 0.167428 Leu + 0.109702 Phe + 0.128822 Lys + 0.048213 His + 0.00015 Trp + 0.282755 Arg + 0.094576 Pro + 0.481984 Glyc3P + 0.015487 DXP.

Fluxes were normalized by setting the Glc-to-G6P reaction to 100 in each strain, providing a common reference for comparing relative carbon allocation within the modeled network. Consequently, differences between *Cz*-WT and *Cz-pkg* reflect differences in flux distribution relative to glucose entry into central metabolism (Table 3). Compared with *Cz-pkg*, *Cz*-WT showed higher normalized flux point estimates for the conversion of PEP to pyruvate (PYR) (r9, 126.2 vs. 105.6) and PYR to acetyl-CoA (AcCoA) (r10, 106.5 vs. 70.5), as well as consistently higher point estimates for the modeled TCA cycle reactions r14–r18. These patterns are consistent with stronger transfer of glucose-derived carbon through pyruvate oxidation and the TCA cycle in *Cz*-WT under 100 mM NaCl. Notably, both PEP-to-OAA (r12, 180.3) and OAA-to-PEP (r13, 185.8) flux point estimates were high in *Cz*-WT. Because these reactions operate in opposite directions in the model, their simultaneous elevation is more conservatively interpreted as enhanced PEP/OAA exchange rather than as an unequivocal increase in net anaplerotic flux. In contrast, *Cz-pkg* showed lower normalized flux point estimates for the reactions from PEP to OAA and AcCoA than *Cz*-WT, indicating a distinct pattern of carbon allocation at the glycolysis–TCA cycle interface. This pattern may be associated with differences in energy and fatty acid metabolism during salinity acclimation.

The central role of the glycolysis–pyruvate–acetyl-CoA interface in salinity-associated carbon redistribution is also supported by studies in other green algae. In *Chlamydomonas* sp. JSC4, dynamic metabolic profiling combined with transcriptional analysis identified pyruvate-to-acetyl-CoA conversion as a rate-limiting step during salinity-induced redistribution of carbon from starch toward lipid synthesis [27]. The higher r10 estimate in *Cz*-WT is therefore consistent with a greater capacity to supply acetyl-CoA for fatty-acid synthesis. Thus, the present ^13^C-MFA results provide flux-level evidence for strain-associated differences in central-carbon throughput, extending the previously reported physiological salt-sensitive phenotype of *Cz-pkg* [24].

### 2.3. Metabolomic Profiling Supports Constraints in Carbon and Redox Metabolism

Metabolite profiling revealed distinct responses over time and between trophic conditions in *Cz*-WT and *Cz-pkg* under 100 mM NaCl treatment. To emphasize the principal biological patterns, representative metabolites associated with central carbon, amino acid, nucleotide, and redox metabolism are summarized in Table 4, whereas the complete metabolite datasets and pathway annotations are provided in Tables S3 and S4.

Several intermediates associated with central carbon metabolism showed broadly similar directional changes in the two strains. Glucose 6-phosphate (G6P) remained below the corresponding control level across all examined conditions, with response ratios of 0.59–0.66 in *Cz-pkg* and 0.56–0.68 in *Cz*-WT. A similar pattern was observed for 6-phosphogluconate (6-PG), which also remained below unity in both genotypes (Table 4, Tables S3 and S4). These changes indicate substantial remodeling of glycolysis/pentose phosphate pathway (PPP)-associated metabolite pools during the response to 100 mM NaCl.

Citrate exhibited a more pronounced strain-specific response that varied with culture condition. In *Cz-pkg*, citrate response ratios ranged from 1.04 to 1.20, whereas in *Cz*-WT the ratio increased to 1.33 after 48 h under mixotrophic conditions (Table 4, Tables S3 and S4). The later increase in citrate in *Cz*-WT was consistent with the higher normalized flux estimates observed for several TCA cycle reactions in the ^13^C-MFA analysis, further indicating a distinct central carbon metabolic response in the wild type under mixotrophic conditions.

Amino-acid-related metabolites also displayed distinct response patterns between the two strains. Argininosuccinate remained below unity in both genotypes and showed particularly low values in *Cz-pkg*, especially at 12 h. In contrast, N-acetyl-L-glutamate and citrulline increased more strongly in *Cz*-WT at later time points under mixotrophic conditions, reaching response ratios of 1.55 and 1.74, respectively, at 48 h (Table 4, Tables S3 and S4). These differences suggest distinct remodeling of arginine and glutamate metabolism between the two strains during salinity acclimation.

Nucleotide-related metabolites showed similarly dynamic responses. dTMP decreased at the earlier sampling points in both strains, followed by an increase at 48 h under mixotrophic conditions, with response ratios of 1.19 in *Cz-pkg* and 1.35 in *Cz*-WT (Table 4, Tables S3 and S4). This temporal pattern indicates that nucleotide metabolism was progressively adjusted during the response to NaCl treatment and was influenced by both genotype and trophic mode.

The NADH pool also showed a marked temporal response to 100 mM NaCl. At 12 h, NADH levels were reduced relative to the corresponding controls in both strains under autotrophic and mixotrophic conditions, with a more pronounced decrease in *Cz*-WT. Under mixotrophic conditions, NADH levels in both strains subsequently recovered toward the control level by 48 h, reaching response ratios of 0.87 in *Cz-pkg* and 0.89 in *Cz*-WT (Table 4, Tables S3 and S4). This transient decrease followed by partial recovery suggests an early perturbation of cellular redox balance and subsequent redox adjustment during salinity acclimation.

Comparable metabolic plasticity has been reported in other green microalgae. In *C. reinhardtii*, salinity exposure alters redox, amino acid, and lipid metabolism [26], while time-resolved metabolite profiling in *Chlamydomonas* sp. JSC4 revealed dynamic changes in metabolite pools and carbon redistribution during salinity acclimation [27]. More recent multi-omics studies in *Chlorella* sp. DT025 and *Scenedesmus obliquus* have further demonstrated coordinated changes in central carbon, nitrogen, lipid, and redox metabolism under salinity treatment [11]. A multi-omics study in *Scenedesmus obliquus* UTEX393 further demonstrated salinity-associated changes in central carbon metabolism, polar metabolites, lipid remodeling, and redox regulation [14]. Together, these findings are consistent with the strain-specific metabolic remodeling observed here and support the combined interpretation of metabolomics and ^13^C-MFA during salinity acclimation.

### 2.4. Transcriptomic Responses Reveal Conserved and Strain-Specific Salinity-Acclimation Programs

To gain deeper insight into transcriptional acclimation to salinity acclimation, we analyzed the transcriptomes of *Cz*-WT and *Cz-pkg* after 24 h of treatment with 100 mM NaCl. Differential expression and gene set enrichment analyses were performed. Venn analysis (Figure 2) showed that 141 genes were significantly upregulated (*p* < 0.05) in both strains. These genes were mainly involved in porphyrin and chloroplast metabolism, glutathione metabolism, glycolysis, gluconeogenesis, amino acid metabolism, terpenoid biosynthesis, photosynthesis, and other key pathways.

Among genes associated with photosynthesis, *por* (Cz06g28160), encoding NADPH-protochlorophyllide oxidoreductase (POR), *petE* (Cz01g35130), encoding plastocyanin, and *pgps* (Cz06g21300), encoding phosphatidylglycerophosphate synthase (PGPS), were significantly upregulated in both strains. Plastocyanin is a copper-containing protein located in the chloroplast lumen that transfers electrons between photosystem I (PSI) and the cytochrome *b_6_f* complex, thereby coupling photosynthesis and aerobic respiration [28]. In the cyanobacterium *Synechocystis* sp. (PCC6803), plastocyanin cooperates with cytochrome c to mediate electron transfer from cytochrome *b6f* to PSI or to cytochrome c oxidase, coupling light energy capture to chemical energy generation [29].

PGPS catalyzes phosphatidylglycerophosphate formation, a committed step in phosphatidylglycerol (PG) biosynthesis. In *C. reinhardtii*, the genes *CrPGP1* and *CrPGP2* have been experimentally demonstrated to encode functional PGPS enzymes, and PG biosynthesis is closely associated with the integrity and function of photosynthetic membrane complexes [30]. Consistently, PG deficiency in *C. reinhardtii* impairs the biogenesis of photosystem II core complexes [31]. The induction of *pgps* in both *Cz*-WT and *Cz-pkg* is therefore consistent with a shared membrane- or thylakoid-lipid acclimation response to salinity.

Conversely, 228 genes were significantly downregulated in both strains (*p* < 0.05). These genes were mainly associated with photosynthesis, MAPK signaling, amino acid and nucleotide metabolism, and transcriptional regulation. The MAPK cascade is an evolutionarily conserved eukaryotic signaling mechanism that transduces external signals through phosphorylation and is commonly involved in responses to drought, high salt, and low-temperature stress in plants [32]. Notably, the transcript encoding a type 2C protein phosphatase (PP2C; Cz09g12010) was downregulated, which may be associated with reduced negative regulation of MAPK signaling during salinity acclimation.

Additionally, the gene *petJ* (Cz01g35120), encoding Cytochrome *c*_6_ (Cyt *c*_6_) in the photosynthetic electron transport chain, was significantly downregulated in both strains. Cyt *c*_6_ is a key electron carrier that shuttles electrons from plastoquinol (via the Cytochrome *b_6_f* complex) to the PSI primary electron acceptor P700, and ultimately to ferredoxin-NADP^+^ reductase (FNR) to produce NADPH [33]. Downregulation of *petJ* suggests that algal cells may be actively modulating electron flow under salinity acclimation to balance energy metabolism.

In summary, under 100 mM NaCl treatment, both *Cz*-WT and *Cz-pkg* exhibit significant transcriptional responses at multiple levels of energy metabolism, photosynthetic electron transport, and signal transduction. Upregulated genes are enriched in functions related to energy conversion and membrane stability (e.g., *pgps*, *petE*), which may help maintain metabolic homeostasis in the wild type under saline conditions. Changes in photosynthetic electron transport and MAPK signaling components reflect adaptive mechanisms of energy redistribution and stress signaling. These results provide a molecular basis for understanding the PKG-mediated regulatory network in salt tolerance.

### 2.5. Differential Expression in Photosynthesis-Related Pathways During Salinity Acclimation

The photosynthetic electron transport chain is tightly coupled to the Calvin–Benson–Bassham (CBB) cycle in microalgae and is essential for maintaining cellular energy supply and carbon fixation [34]. The light reactions not only drive electron flow and ATP synthesis but also provide carbon skeletons and reducing power under both autotrophic and mixotrophic conditions [35]. Therefore, to investigate energy metabolism and photosynthetic carbon fixation under salinity acclimation, we systematically analyzed transcriptional changes in key photosynthetic genes after 100 mM NaCl treatment.

Salinity treatment has a dual effect on microalgal photosystems. Low salinity can stimulate photosynthetic electron transport and increase energy metabolism, whereas high salinity can induce ROS accumulation, damaging photosystem complexes and inhibiting the light reactions [9]. Under 100 mM NaCl treatment, most genes related to photosystem I (PSI) and photosystem II (PSII) in *Cz*-WT were significantly upregulated under autotrophic conditions (Figure 3b), indicating a coordinated response of the photosynthetic apparatus to salinity acclimation. However, under mixotrophic conditions (glucose present), many photosynthesis-related genes were significantly downregulated during the first 6 h of salt treatment (Figure 3b), indicating metabolic repression of photosynthesis by exogenous organic carbon.

In contrast, *Cz-pkg* under autotrophic conditions exhibited a similar trend to *Cz*-WT; photosystem-related genes were broadly upregulated, with most showing significant increases at 12 h and 24 h of treatment, except for the photosystem II protein Q1 (PsbQ1), which remained continuously downregulated (Figure 3a). *Cz-pkg* showed a broadly similar pattern under autotrophic conditions, but its transcriptional response was weaker under mixotrophic conditions, and the transcript encoding PsbQ1 remained downregulated throughout the examined time points (Figure 3a), indicating a less coordinated transcriptional response of the photosynthetic apparatus under elevated salinity.

Additionally, analysis of genes involved in chlorophyll metabolism [22] showed that under 100 mM NaCl, both *Cz*-WT and *Cz-pkg* upregulated genes involved in chlorophyll biosynthesis under autotrophy. In *Cz*-WT, the transcript encoding light-dependent protochlorophyllide oxidoreductase (POR) increased 5.71-fold, while the transcript encoding magnesium-protoporphyrin IX methyltransferase (CHLM) increased 10.42-fold, indicating enhanced transcription of genes involved in chlorophyll biosynthesis. This correlates with the growth stimulation of *Cz*-WT under 100 mM NaCl. A similar trend was observed in *Cz-pkg* under autotrophy, with CHLM and POR upregulated (4.72- and 2.12-fold, respectively) at 6 h, indicating preserved adaptability of the chlorophyll biosynthetic system in the absence of PKG function.

Overall, under autotrophic conditions, many transcripts associated with PSI and PSII increased in *Cz*-WT after treatment with 100 mM NaCl, whereas under mixotrophic conditions, numerous genes involved in photosynthesis were downregulated during the early response (Figure 3b). *Cz-pkg* showed a broadly similar response under autotrophy conditions, but its transcriptional response was weaker under mixotrophy, and the transcript encoding PsbQ1 remained downregulated throughout the examined time points (Figure 3a). These results indicate that the transcriptional acclimation of the photosynthetic apparatus varies with both strain background and trophic mode.

### 2.6. Differential Expression of ROS Scavenging Genes Under Salinity Acclimation

Salt stress induces rapid ROS generation in plant and algal cells. High levels of ROS can cause lipid peroxidation, protein inactivation, and DNA damage. However, ROS also function as signaling molecules involved in various stress response pathways and gene regulation [36]. To prevent oxidative damage, algal cells rely on both enzymatic and non-enzymatic antioxidant systems to maintain redox homeostasis [37]. Key antioxidant enzymes include superoxide dismutases (SODs), catalase (CAT), ascorbate peroxidase (APX), glutathione peroxidase (GPX), and glutathione reductase (GR) [38]. SOD converts superoxide anions (O_2_^−^) to H_2_O_2_ and is the first line of defense. Depending on metal cofactors, there are three types of SOD: Fe-SOD (mainly chloroplastic), Mn-SOD (mitochondrial and peroxisomal), and Cu/Zn-SOD (cytosolic and chloroplastic). These isoforms together enable a multi-level defense across cellular compartments [39].

As shown in Figure 4, under 100 mM NaCl treatment, multiple ROS-scavenging genes in *Cz*-WT were significantly upregulated under both autotrophic and mixotrophic conditions. Transcripts encoding Fe-SOD, Mn-SOD, and Cu/Zn-SOD increased markedly, while genes encoding APX, GPX, and GR were also generally induced. For example, the transcript level of the gene encoding GR increased 4.83-fold at 6, indicating a rapid transcriptional adjustment of antioxidant-related pathways in *Cz*-WT during salinity acclimation. In contrast, the transcriptional response in *Cz-pkg* was less pronounced. Genes encoding SODs showed no significant induction, and several genes encoding GPX, GR, and APX isoforms were downregulated under mixotrophic conditions. These contrasting expression patterns indicate that the two strains differ substantially in the magnitude and coordination of antioxidant-related transcriptional responses to salinity acclimation.

Together, these results show that *Cz*-WT and *Cz-pkg* exhibit distinct transcriptional responses involving antioxidant pathways during salinity acclimation. The coordinated induction of genes associated with SODs and glutathione metabolism in *Cz*-WT is consistent with a broader transcriptional adjustment related to redox homeostasis, whereas *Cz-pkg* shows a weaker and less coordinated response, particularly under mixotrophic conditions. When considered together with the differences observed in central carbon metabolism, these transcriptional patterns suggest that regulation of redox processes contributes to the broader metabolic divergence between the two strains under salinity acclimation.

### 2.7. Integrated Metabolic Interpretation of Salinity Acclimation

Central carbon metabolism (CCM) is the core hub of microalgal metabolic activity, encompassing the Calvin cycle, glycolysis/gluconeogenesis, the TCA cycle, and the PPP [40]. These pathways not only provide energy and reduce power for cell growth but also supply carbon skeletons for the biosynthesis of lipids, amino acids, and pigments. To determine whether the ^13^C-MFA-derived flux changes were supported at the transcriptional level, we analyzed time-course expression profiles of genes associated with the Calvin cycle, glycolysis/gluconeogenesis, starch metabolism, and the TCA cycle under 100 mM NaCl treatment (Figure 5, Figure 6, Figure 7 and Figure 8).

#### 2.7.1. Calvin Cycle

The Calvin cycle is the core pathway of photosynthetic carbon fixation, where ribulose-1,5-bisphosphate carboxylase/oxygenase (RuBisCO) catalyzes the conversion of CO_2_ to form 3-phosphoglycerate (PGA), providing primary carbon skeletons for anabolism [41]. Our results (Figure 5) showed that under 100 mM NaCl, the Rubisco small subunit gene (*RbcS*) in *Cz-pkg* did not change significantly under either autotrophic or mixotrophic conditions. However, in *Cz*-WT under mixotrophy, *RbcS* was considerably downregulated, indicating a reduced transcriptional response of the RuBisCO small subunit during salinity acclimation. Additionally, fructose-1,6-bisphosphatase (FBPase3) was significantly upregulated in *Cz-pkg* under autotrophic conditions but downregulated under mixotrophic conditions, suggesting that the transcriptional response of the Calvin cycle in *Cz-pkg* varies with trophic mode and may be influenced by the presence of exogenous glucose.

#### 2.7.2. Glycolysis (EMP Pathway) and Gluconeogenesis

Glycolysis is the primary pathway for energy production and carbon skeleton supply, and it is present in both the chloroplast and cytosol of *C. zofingiensis*, similar to *Chlamydomonas* and *Nannochloropsis* [5]. The key rate-limiting enzymes are hexokinase (HXK), phosphofructokinase (PFK), and pyruvate kinase (PK). In this study, glycolysis specifically refers to the Embden–Meyerhof–Parnas (EMP) pathway from glucose to pyruvate. Figure 6 showed that transcripts encoding PFK were significantly upregulated in *Cz*-*pkg*. In contrast, transcripts encoding both chloroplastic and cytosolic PK isoforms were downregulated, suggesting a weaker transcriptional capacity for the final step of glycolysis in the mutant and potentially affecting pyruvate supply for downstream acetyl-CoA formation. By contrast, previous studies have shown that under 200 mM NaCl, *Cz*-WT significantly upregulates PK (over 30-fold) [21], further confirming the mutant’s apparent defect in glycolytic response.

Gluconeogenesis is the reverse pathway of glycolysis, involving enzymes such as pyruvate carboxylase (PYC), phosphoenolpyruvate carboxykinase (PEPCK), fructose-1,6-bisphosphatase (FBP), and glucose-6-phosphatase, which synthesize glucose precursors from non-sugar sources [42,43]. In *Cz-pkg* under autotrophy, FBPase III was significantly upregulated. In contrast, under mixotrophic conditions, FBPase II was upregulated and FBPase III was downregulated, suggesting that the transcriptional regulation of gluconeogenesis varies with trophic mode. PEPCK, which catalyzes the conversion of OAA to phosphoenolpyruvate, also showed opposite expression trends under autotrophic and mixotrophic conditions in *Cz-pkg*. In *Cz*-WT, genes associated with glycolysis and gluconeogenesis showed a different regulatory pattern. Consistent with these transcriptional differences, the normalized ^13^C-MFA point estimates for the PEP-to-PYR and PYR-to-AcCoA reactions were higher in *Cz*-WT than in *Cz-pkg*. Together, these results indicate distinct regulation of carbon allocation around the PEP–pyruvate node between the two strains during salinity acclimation.

#### 2.7.3. Pentose Phosphate Pathway (PPP)

The PPP not only produces intermediates for carbohydrate metabolism but also supplies NADPH needed for lipid and carotenoid synthesis [44]. The key enzymes include glucose-6-phosphate dehydrogenase (G6PDH; Cz06g12080 and Cz03g12030), 6-phosphogluconolactonase (PGLS; Cz04g18010 and Cz11g03250), and 6-phosphogluconate dehydrogenase (6PGDH; Cz05g06160). We found that in *Cz-pkg*, the transcript encoding PGLS was significantly upregulated at 12 h, and the transcript encoding 6PGDH was upregulated at 24 h, indicating a temporal transcriptional adjustment of the PPP. In *Cz*-WT, transcripts encoding G6PDH and PGLS were generally downregulated, whereas the transcript encoding 6PGDH increased under mixotrophic conditions, differing from the strong upregulation observed under 200 mM NaCl in our previous work [21]. Together, these results indicate distinct transcriptional regulation of the PPP between the two strains during salinity acclimation, while the modeled oxidative PPP flux estimates remained similar (Table 3).

#### 2.7.4. Starch Metabolism

Starch is the primary carbon reserve in algal cells, and its breakdown under stress provides carbon and energy for lipid and secondary metabolite synthesis [45]. Under external stress, excess carbon skeletons are often redirected from starch to lipids, resulting in starch degradation and lipid accumulation, making triacylglycerol the preferred storage form [44]. From an energy perspective, the ATP yield per carbon is higher for fatty acid oxidation (~6.7 ATP per carbon for palmitate) than for glucose oxidation via glycolysis and the TCA cycle (~5 ATP per carbon) [46]. Studies have shown that salinity treatment can promote conversion of starch to lipids in various algae (e.g., *Chlamydomonas* sp. [47], *Micractinium inermum* [48], and *Chlorella sorokiniana* [49]). However, the exact mechanism is unclear. Under 100 mM NaCl treatment (Figure 7), *Cz-pkg* under autotrophic conditions showed significant decreases in transcripts encoding starch synthases (SSS and GBSS), suggesting a weaker transcriptional response associated with starch synthesis. Meanwhile, genes involved in starch degradation, including those encoding SPL and AMY, showed variable expression among different isoforms. Under mixotrophic conditions, no significant changes were observed in genes associated with starch synthesis, whereas several genes involved in starch degradation were upregulated. In contrast, *Cz*-WT showed increased expression of several genes associated with starch synthesis under both trophic conditions, together with reduced expression of genes involved in starch degradation under autotrophic conditions. These transcriptional patterns suggest distinct regulation of starch metabolism between the two strains during salinity acclimation. Notably, *Cz*-WT also showed increased biomass accumulation under 100 mM NaCl treatment.

#### 2.7.5. TCA Cycle

The TCA cycle serves as a central hub that links carbohydrate, lipid, and amino acid metabolism and is crucial for energy supply and precursor generation [50]. Our results (Figure 8) show that in *Cz-pkg*, the transcript encoding citrate synthase (CIS1) was significantly upregulated at 12 h, and the transcript encoding succinate dehydrogenase (SDH1) was downregulated considerably under autotrophy (while under mixotrophy, the transcript encoding SDH1 showed an initial increase followed by a decrease). In *Cz*-WT, transcripts encoding CIS1 and SDH2 were downregulated, whereas the transcript encoding malate dehydrogenase (MDH) was significantly upregulated under both conditions. These expression patterns indicate differences in the transcriptional regulation of TCA cycle metabolism between the two strains and trophic conditions. Under mixotrophy, transcripts encoding two isoforms of α-ketoglutarate dehydrogenase (OGDH2 and OGDH3) showed opposite trends (OGDH2 down, OGDH3 up) in *Cz*-WT, and the transcript encoding succinyl-CoA synthetase (SCS) was downregulated.

Taken together, *Cz-pkg* showed lower normalized flux point estimates for several reactions connecting glycolysis, pyruvate oxidation, and the TCA cycle, together with distinct transcriptional changes in the PPP and TCA cycle. In comparison, *Cz*-WT showed higher normalized flux point estimates for pyruvate-to-acetyl-CoA conversion and several modeled reactions of the TCA cycle. These results indicate distinct patterns of central carbon allocation between the two strains during salinity acclimation. Ion homeostasis is also an important component of salinity acclimation. However, the present study did not include direct measurements of intracellular Na^+^/K^+^ levels or a targeted functional analysis of ion transporters and channels. Therefore, this aspect was not interpreted mechanistically here and warrants dedicated investigation in future studies.

## 3. Materials and Methods

### 3.1. Algal Strains and Culture Conditions

The wild-type strain of *C. zofingiensis* (*Cz*-WT, ATCC 30412) was obtained from the American Type Culture Collection (ATCC). The mutant strain (*Cz-pkg*) was generated from the wild-type strain through chemical mutagenesis using MNNG, as previously described [22]. Both strains were maintained on solid Kuhl medium (pH 6.5), as previously described [22], and liquid cultures were inoculated into flasks containing Kuhl medium. The composition of Kuhl medium was as follows: KNO_3_ 1.01 g/L, NaH_2_PO_4_·H_2_O 0.62 g/L, Na_2_HPO_4_·2H_2_O 0.089 g/L, MgSO_4_·7H_2_O 0.247 g/L, CaCl_2_·2H_2_O 14.7 mg/L, Na_2_EDTA·H_2_O 6.95 mg/L, FeSO_4_·7H_2_O 6.95 mg/L, H_3_BO_3_ 0.061 mg/L, MnSO_4_·H_2_O 0.169 mg/L, ZnSO_4_·7H_2_O 0.287 mg/L, CuSO_4_·5H_2_O 0.0025 mg/L, (NH_4_)_6_Mo_7_O_24_·4H_2_O 0.01235 mg/L. The medium was adjusted to pH 6.5 with 2 M NaOH and sterilized.

Cultures were incubated at 25 °C under continuous white light at a photosynthetic photon flux density (PPFD) of 30 μmol photons m^−2^ s^−1^, with shaking at 150 rpm. For mixotrophic conditions, glucose was added at a final concentration of 5 g/L. Salinity treatments were carried out by adding NaCl to the above medium at final concentrations of 0, 25, 50, 100, 150, or 200 mM. For ^13^C-labeling experiments, the carbon sources were (1) 1-^13^C-glucose (5 g/L, 100% labeled) and (2) a mixture of U-^13^C-glucose (1 g/L) and unlabeled glucose (4 g/L, total 5 g/L, 20% labeled). All other medium components were identical to those used for the standard Kuhl medium.

### 3.2. Biomass Determination

The biomass concentration was determined by dry weight. A 3 mL aliquot of the log-phase culture was centrifuged at 5000 rpm for 5 min at room temperature, and the pellet was washed twice with deionized water to remove residual medium. The cells were filtered through a pre-dried and pre-weighed filter paper (mass = *m*_1_). The filter paper and cells were dried at 80 °C under vacuum to a constant weight and weighed (mass = *m*_2_). The dry cell weight (DCW, g/L) was calculated as

(3) DCW (g/L) = (*m*_2_ − *m*_1_)/*V*,

where *V* denotes the volume (L) of culture used for filtration. Measurements were repeated three times and reported as the mean ± standard deviation.

### 3.3. Carbohydrate Content Determination

The carbohydrate content was measured using the phenol–sulfuric acid method [51,52]. Briefly, 1 mL of culture (with a known DCW) was centrifuged at 5000 rpm for 5 min at room temperature. The pellet was resuspended in 500 µL of ice-cold acetic acid and heated at 80 °C for 20 min to lyse the cells. After cooling, 1 mL of acetone was added. The mixture was then centrifuged at 5000 rpm for 10 min at room temperature, and the supernatant was discarded. We added 0.25 mL of 4 M trifluoroacetic acid (TFA) to resuspend the precipitate and incubated the suspension at 100 °C for four hours to hydrolyze carbohydrates to monosaccharides. After cooling to room temperature, the mixture was centrifuged at 13,000 rpm for 5 min, and the supernatant was then collected. Glucose standards and a reagent blank were processed in parallel with the samples to establish the calibration curve and correct for background absorbance, respectively. Absorbance was measured at 490 nm (*A*_490_), and the carbohydrate concentration was calculated according to Equation (4):

(4) Carbohydrate concentration (g/L) = 8.1495 × *A*_490_ − 0.8295

### 3.4. Lipid Content and Fatty Acid Composition Determination

Lipid content and fatty acid methyl ester (FAME) profiles were determined using GC-MS. Approximately 20 mg of freeze-dried algal biomass was weighed into a screw-cap glass vial, to which 2 mL of working solution I (methanol:sulfuric acid = 1000:1), containing 0.05% butylated hydroxytoluene (BHT) and 0.5 mL of internal standard solution (1 mg/mL heptadecanoic acid in hexane), and 1 mL of toluene were added. The mixture was vortexed and heated overnight at 50 °C to perform in situ transesterification. After cooling to room temperature, 1 mL of a 7.5 g/L NaHCO_3_ solution was added, and then 2 mL of hexane was added and mixed. The mixture was allowed to stand for phase separation, and the upper hexane layer containing the FAMEs was collected. The extract was centrifuged at 12,000× *g* for 10 min to remove particulates, and the supernatant was analyzed by GC-MS. GC-MS was performed on an Agilent 7890B–5977B system (Agilent Technologies, Inc., Santa Clara, CA, USA) equipped with a DB-WAX column (30 m × 0.25 mm × 0.25 µm). The injector and ion-source temperatures were 240 and 200 °C, respectively. Helium was used as the carrier gas at 1.2 mL/min, with an injection volume of 1 µL and a split ratio of 5:1. The mass spectrometer was operated in electron ionization (EI) mode at 70 eV with a full-scan range of m/z 50–650. Individual FAMEs were identified by comparison of their retention times with FAME standards and further confirmed by matching their mass spectra against the NIST mass spectral library. Quantification was based on peak areas relative to the internal standard. Fatty acid composition was expressed as mass% of total fatty acids.

### 3.5. Protein Content Determination

The protein content was measured using a bicinchoninic acid (BCA) Protein Assay Kit (TaKaRa Bio Inc., Kusatsu, Shiga, Japan; T9300A) according to the manufacturer’s instructions, with bovine serum albumin (BSA) as the standard. A 1 mL aliquot of log-phase culture was centrifuged at 5000 rpm for 5 min, and the pellet was washed twice with deionized water. The pellet was then resuspended in 500 µL of 2 M NaOH and heated at 80 °C for 10 min to solubilize the proteins. After cooling to room temperature, 1 mL of deionized water was added, and the suspension was centrifuged at 13,000 rpm for 30 min. The supernatant was then collected. This extraction was repeated three times, and the supernatants were combined. BSA standards and a reagent blank were processed in parallel with the samples to establish the calibration curve and correct for background absorbance, respectively. Absorbance was measured at 562 nm (*A*_562_), and the protein concentration was calculated according to Equation (5):

(5) Protein concentration (mg/L) = 1493.9 × *A*_562_ − 139.07

### 3.6. Amino Acid Composition Analysis

The acid-hydrolysable amino acid composition of freeze-dried biomass was determined using Waters AccQ•Tag chemistry. The 17 amino acids analyzed corresponded to the analytes included in the calibration mixture used in this study and constituted the targeted amino acid panel for the acid-hydrolysate analysis. Because conventional HCl hydrolysis does not allow all amino acids to be recovered quantitatively, the resulting values represent the amino acids recovered under the specified hydrolysis conditions rather than a complete absolute amino acid profile. In particular, Asn and Gln are converted to Asp and Glu, respectively, during acid hydrolysis, whereas the recoveries of some acid-labile amino acids may also be affected by the hydrolysis procedure. The detailed protocol is as follows:

(1) Sample preparation: Approximately 50 mg of freeze-dried algae powder was weighed into a 2 mL centrifuge tube with four stainless-steel beads. The sample was disrupted using a bead beater at 30 Hz for 10 min (three cycles) under cold conditions, until the cells turned white. Then, 1 mL of precooled 6 M HCl was added, and the sample was vortexed for an additional 10 min to ensure thorough lysis. The slurry was transferred to a 15 mL glass screw-cap bottle using an additional 4 mL of 6 M HCl. The bottle was flushed with nitrogen gas to remove oxygen and then sealed again. The sample was hydrolyzed at 110 °C in an oven for 24 h. After cooling to room temperature, the pH was adjusted to 5–7 using a saturated solution of Na_2_CO_3_. The hydrolysate was filtered through a qualitative filter paper into a volumetric flask and diluted to 25 mL with ultrapure water. The solution was then filtered through a 0.22 µm membrane before analysis.

(2) Derivatization: A calibration mixture containing the 17 targeted amino acids was prepared at concentrations of 25, 50, 100, 250, and 500 pmol/µL for the calibration curves. For each sample or standard, 10 µL was pipetted into an AccQ•Tag derivatization vial, followed by the addition of 70 µL AccQ•Fluor borate buffer (Buffer 1) and 20 µL AccQ•Fluor reagent. The mixture was vortexed for 15 s and incubated at room temperature for 1 min. The vial was then sealed and heated at 55 °C for 10 min to complete the derivatization. A blank was prepared by mixing 80 µL borate buffer with 20 µL derivatization reagent. All pipettes and containers were predried in an oven before use to avoid moisture.

(3) HPLC analysis: Derivatized samples were analyzed on a Waters AccQ•Tag HPLC column. Mobile phase A was AccQ-Tag Eluent A (diluted 1:9 with water), mobile phase B was acetonitrile, and mobile phase C was water. The injection volume was 10 µL, the column temperature was 37 °C, and the flow rate was 1.0 mL/min. The gradient program is given in Table S5. Amino acids were identified and quantified based on retention times and calibration curves.

### 3.7. DNA and RNA Content Determination

#### 3.7.1. DNA Content

Ten milliliters of log-phase culture was centrifuged at 3000 rpm for 5 min at 4 °C in DNase/RNase-free centrifuge tubes to harvest cells. The pellet was washed twice with 2 mL of 1 M MgCl_2_. Then, 5 mL of ice-cold 0.25 M perchloric acid (HClO_4_) was added, and the suspension was incubated on ice for 30 min. The mixture was centrifuged to remove the supernatant, and the precipitate was resuspended in 4 mL 0.5 M HClO_4_. The sample was then incubated at 70 °C for 15 min and centrifuged to collect the supernatant. The extraction with 0.5 M HClO_4_ (4 mL) was repeated twice more, and all supernatants were combined. RNase (20 µL) was added, and the mixture was incubated at 37 °C for 10 min to remove RNA. Then, the volume was adjusted to 10 mL with sterile water, and the mixture was centrifuged at 10,000 rpm for 10 min to remove debris. DNA content was measured using an NP80 ultra-microUV spectrophotometer (Implen GmbH, Munich, Germany).

#### 3.7.2. RNA Content

RNA content was measured using a procedure similar to that used for determining DNA content. A 10 mL culture sample was centrifuged as above. The pellet was washed three times with 400 µL 0.7 M perchloric acid. Then, 400 µL 0.3 M KOH was added, and the sample was heated at 37 °C for 60 min to lyse the cells. After cooling, 133 µL of 3 M perchloric acid was added, and the mixture was mixed. The mixture was then centrifuged, and the supernatant was collected. The pellet was treated twice more with 0.5 M perchloric acid and centrifuged. The combined supernatants were treated with 20 µL DNase at 37 °C for 10 min to remove DNA. The volume was adjusted to 2 mL, and then the sample was centrifuged at 10,000 rpm for 10 min to remove debris. RNA content in the supernatant was measured with the NP80 spectrophotometer. All steps were performed under sterile conditions to avoid contamination.

### 3.8. Metabolite Extraction and Untargeted Metabolomics

For untargeted metabolomics analysis, cells of *Cz*-WT and *Cz-pkg* were harvested at 12 h and 48 h following treatment with 100 mM NaCl. A 50 mL culture was centrifuged at 1000 rpm for 5 min at 4 °C. The pellet was washed with deionized water and immediately frozen in liquid nitrogen to rapidly halt metabolic activity. Cells were resuspended in 80% methanol (pre-cooled to −80 °C) and disrupted by two rounds of bead beating (full speed, 10 min each) at low temperature. After centrifuging, the supernatant was collected. The cell pellet was re-extracted once with 80% methanol, and the supernatants were pooled. LC-grade dichloromethane was added to the combined extract to remove pigments, and then the extract was concentrated under vacuum. The concentrate was resuspended in aqueous methanol and analyzed using ultra-performance liquid chromatography coupled with tandem mass spectrometry (UPLC-MS/MS; Waters ACQUITY UPLC H-CLASS PLUS/XEVO TQ-XS) in both positive and negative ion modes.

Untargeted metabolomics and data processing were performed by Majorbio Bio-Pharm Technology Co., Ltd. (Shanghai, China). Pooled quality-control (QC) samples were prepared by mixing equal aliquots of all analytical samples and were interspersed throughout the analytical sequence to monitor analytical reproducibility. Data preprocessing was performed using the Majorbio Cloud Platform (https://cloud.majorbio.com/; accessed on 23 June 2021). Metabolic features detected in at least 80% of samples in any group were retained, and remaining missing values were imputed with the minimum value. Peak intensities were normalized by total-sum normalization, variables with an relative standard deviation (RSD) > 30% in the QC samples were removed, and the resulting data were log_10_-transformed before statistical analysis [53]. Metabolites were annotated by MS/MS spectral matching against in-house and public/theoretical libraries and classified according to the Majorbio B(i)/B(ii) confidence system. Metabolite data processing, annotation, and differential-metabolite analysis were performed using the Majorbio Cloud Platform (https://cloud.majorbio.com/). Differential metabolites were defined by variable importance in projection (VIP) > 1 and *p* < 0.05, fold-change and false discovery rate (FDR) values were also calculated.

### 3.9. ^13^C-Labelled Amino-Acid Enrichment

For ^13^C labeling, cells (at ~1% inoculum) were transferred into medium containing 20% [U-^13^C] glucose and grown at 25 °C and 300 rpm until the optical density at 540 nm (OD_540_) reached ~1. The cells were then harvested and washed twice with 0.9% NaCl. Pellets were hydrolyzed in 1.5 mL 6 M HCl at 110 °C for 24 h and then dried at 80 °C. The dried hydrolysate was dissolved in 100 µL of tetrahydrofuran and derivatized with 50 µL of TBDMS triflate reagent at 85 °C for 1 h. GC-MS then quantified the 13C abundance of individual amino acids. The mass isotopomer distributions (MDVs) of selected TBDMS-derived amino acid fragments were calculated from the GC–MS ion intensities. The measured MDVs were corrected for the natural abundances of stable isotopes contributed by C, H, O, N, Si, and S, including isotope contributions introduced during derivatization, and were subsequently normalized so that the sum of all isotopomer fractions equaled 1. The fractional labeling of the analyzed fragments was compared with the enrichment of the labeled glucose substrate as a quality check. Low-intensity or poorly resolved fragment signals were treated cautiously because they may introduce greater uncertainty into the calculated MDVs. The corrected amino acid MDVs were then used to derive the corresponding precursor-metabolite MDVs for subsequent metabolic flux analysis. For carbon fixation by CO_2_, the apparent labeling of CO_2_ was corrected using [1-^13^C]-glucose (100% labeled) as a reference. Three independent biological replicates were analyzed for each isotope-labeling condition. The corrected mass isotopomer distributions obtained from the three biological replicates were averaged and used as isotopic constraints for subsequent metabolic flux analysis.

### 3.10. Metabolic Network Construction

A metabolic network for *C. zofingiensis* was constructed based on our previous work [54], encompassing major pathways including the EMP glycolytic pathway, PPP, TCA cycle, glyoxylate cycle, amphibolic pathway, synthesis of fatty acids, amino acids and nucleotides, oxidative phosphorylation, transhydrogenation, transport processes, and the MEP pathway for carotenoid synthesis. This network was used for ^13^C-MFA simulations and flux calculations. Based on fluxes through each path, we inferred the distribution of carbon skeletons under different conditions. Detailed reactions are listed in Appendix A.

### 3.11. Transcriptome Sequencing and Analysis

To compare the transcriptional response of *Cz*-WT and *Cz-pkg* to salinity acclimation, samples were collected at 0 h, 6 h, 12 h, and 24 h after 100 mM NaCl treatment under both autotrophic and mixotrophic (5 g/L glucose) conditions. Cells were rapidly frozen in liquid nitrogen and stored at −80 °C. RNA extraction and quality control were performed by Majorbio Bio-pharm Technology Co., Ltd. (Shanghai, China). To prevent genomic DNA contamination, the extracted RNA was treated with RNase-free DNase I (TaKaRa). The concentration and purity were then assessed using a NanoDrop 2000 spectrophotometer (Thermo Fisher Scientific, Wilmington, DE, USA). RNA integrity was evaluated by electrophoresis on a 1% agarose gel stained with GelRed, and the RNA bands were visualized under UV illumination using a Gel Doc XR + Imaging System (Bio-Rad, Hercules, CA, USA). Sequencing libraries were prepared and sequenced on the Illumina NovaSeq 6000 platform (Illumina, San Diego, CA, USA).

Raw sequencing reads were quality-filtered using SeqPrep (https://github.com/jstjohn/SeqPrep, accessed on 21 June 2021) to obtain clean reads in FASTQ format. Clean reads were aligned to the published *C. zofingiensis* reference genome [15] using Hisat2 and Bowtie2 to obtain mapped reads for transcript assembly and quantification. Gene expression levels were calculated as FPKM (Fragments Per Kilobase of transcript per Million mapped reads) using RSEM v1.3.1. Shared and unique expressed genes between samples were identified by Venn analysis. Differential expression analysis was performed using DESeq2 based on raw read counts [55]. Differentially expressed genes (DEGs) were identified by comparing each treatment group with the 0 h control. Genes with an absolute log_2_ fold change (|log_2_FC|) ≥ 1 and an adjusted *p*-value < 0.05 were considered significantly differentially expressed. Each treatment had three biological replicates to ensure reliability and reproducibility. DEGs in pathways of interest were functionally annotated and subjected to enrichment analysis. Gene Ontology (GO) categorization was based on the GO database (http://www.geneontology.org/), and KEGG pathway analysis was performed using the Kyoto Encyclopedia of Genes and Genomes (KEGG, http://www.genome.jp/kegg/, accessed on 21 June 2021). Enriched GO terms or KEGG pathways with adjusted *p*-value < 0.05 (*p* < 0.05) were considered statistically significant.

### 3.12. Data Analysis

For endpoint comparisons among multiple NaCl concentrations within each strain, one-way ANOVA followed by Tukey’s multiple-comparisons test was performed using GraphPad Prism version 9.0 (GraphPad Software, San Diego, CA, USA), with *p* < 0.05 considered statistically significant. Unless otherwise stated, data from three independent biological replicates are presented as mean ± standard deviation. For ^13^C-MFA, corrected isotopomer data from three independent biological replicates were averaged before analysis with Metran. Precursor-metabolite MDVs and intracellular flux distributions were estimated by least-squares fitting. Fluxes were normalized by setting the glucose-to-G6P flux to 100 and are reported as relative point estimate. Because replicate-specific flux estimates and formal nonlinear confidence intervals were not generated in the original analysis, no statistical significance was assigned to differences in flux estimates between *Cz*-WT and *Cz-pkg*.

## 4. Conclusions

Integrated ^13^C-MFA, metabolite profiling, and time-series transcriptomics indicate that *Cz*-WT maintains higher normalized flux estimates through the glycolysis–pyruvate–acetyl-CoA/TCA interface and a more coordinated photosynthetic and antioxidant transcriptional response than the salt-sensitive *Cz-pkg* mutant under 100 mM NaCl. Rather than re-establishing the salt-sensitive phenotype previously reported for the same strain pair [24], the principal advance of the present study is the flux-level localization of strain-associated metabolic constraints at the glycolysis–TCA interface. Metabolite profiling further reveals broad salinity-responsive remodeling in both genotypes, while the shared induction of *pgps* is consistent with membrane-lipid acclimation. Collectively, these results identify central-carbon throughput and energy–redox coordination as metabolic features associated with differential salinity acclimation in *C. zofingiensis*. Direct PKG substrates and causal phosphorylation events remain to be established through genetic complementation, phosphoproteomics, kinase assays, and biochemical target validation. From an applied perspective, these findings may inform strain selection and metabolic-engineering strategies to improve salinity robustness in saline or seawater-based microalgal production systems, with the broader goal of reducing dependence on freshwater resources.

## Figures and Tables

**Figure 1:**
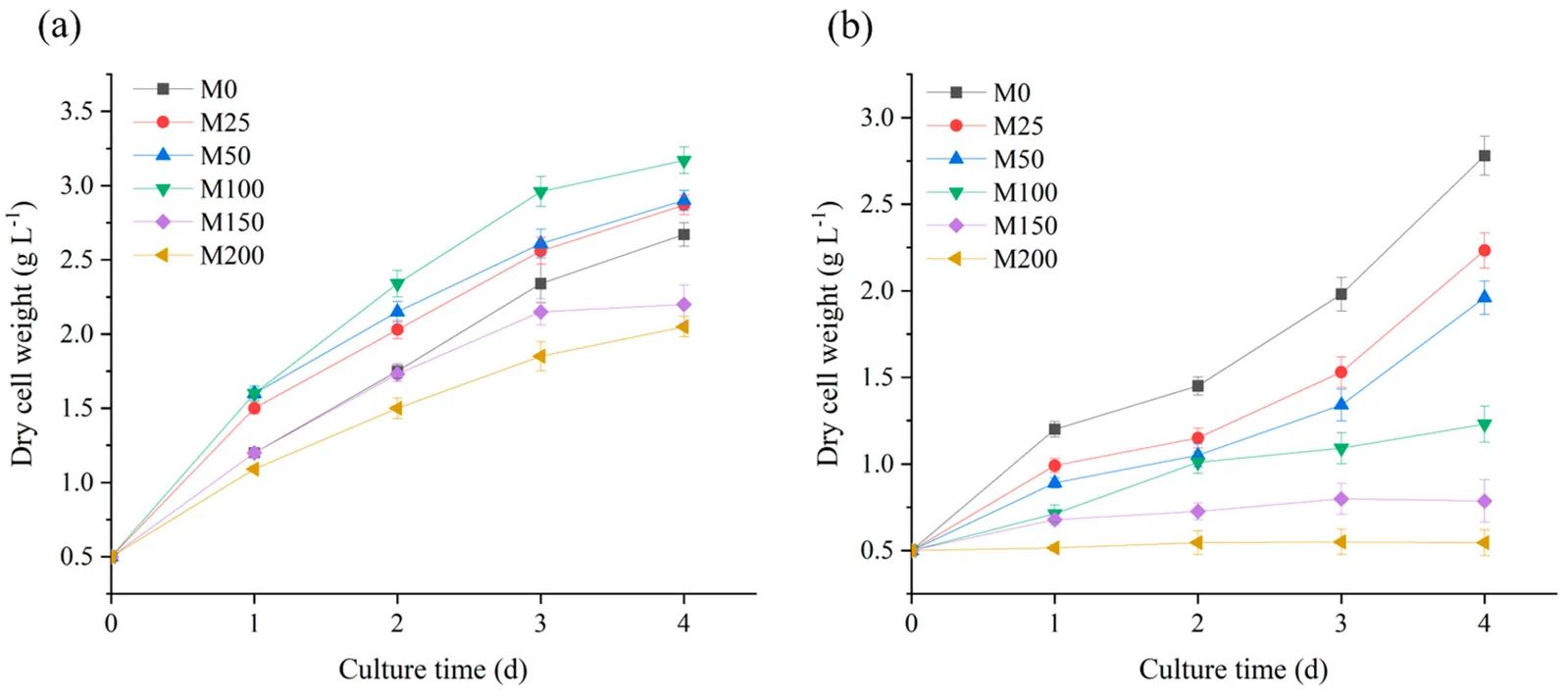
Effects of different NaCl concentrations on the biomass accumulation of *C. zofingiensis* under mixotrophic conditions (5 g/L glucose). (**a**) Biomass of wild type (*Cz*-WT), (**b**) biomass of mutant (*Cz-pkg*). M0-M200 indicated different salt treatment concentrations under mixotrophic conditions (5 g/L glucose).

**Figure 2:**
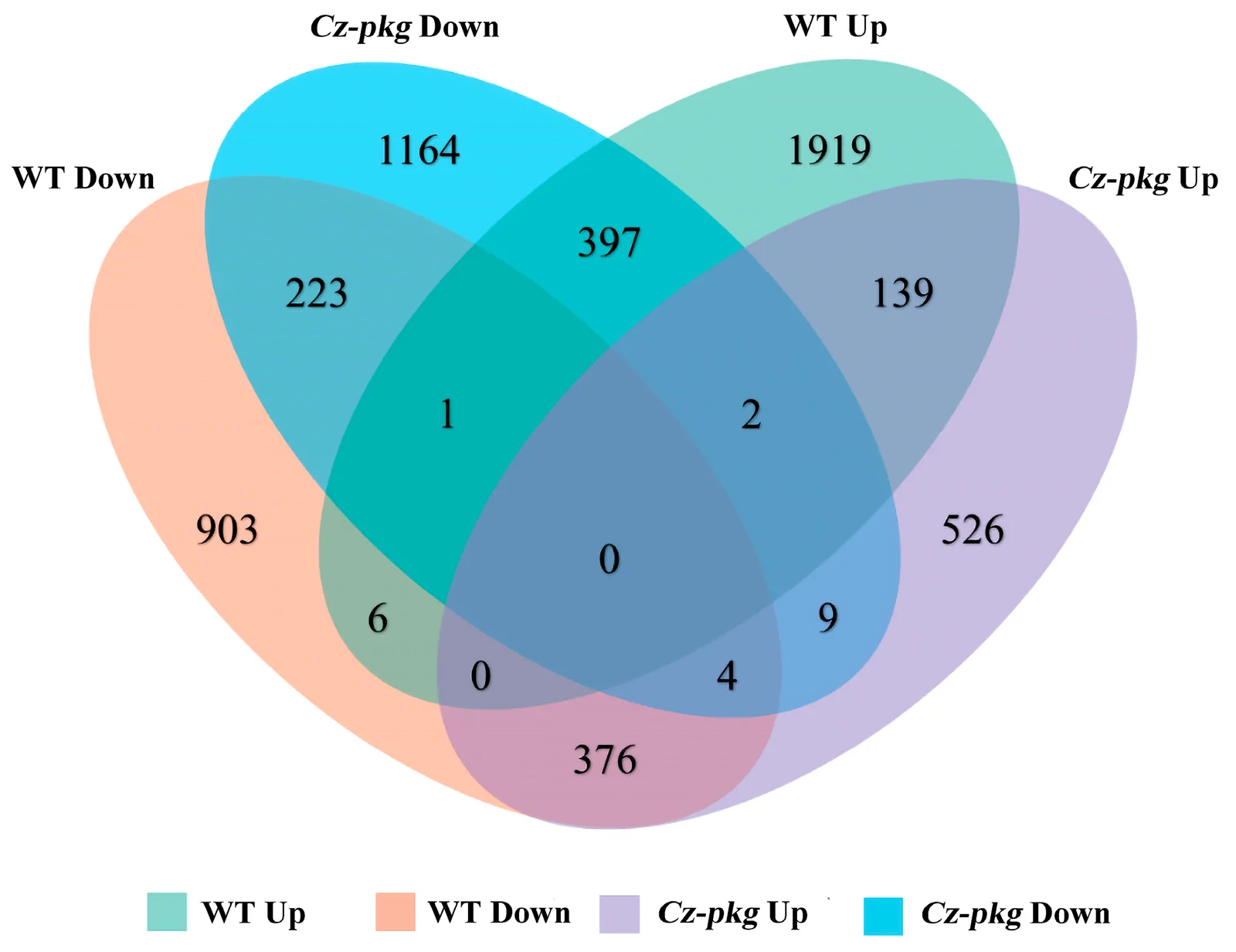
Venn analysis of differentially expressed gene sets under 100 mM NaCl treatment. Shared genes (center intersection) are significantly upregulated in both *Cz*-WT and *Cz-pkg* (*p* < 0.05).

**Figure 3:**
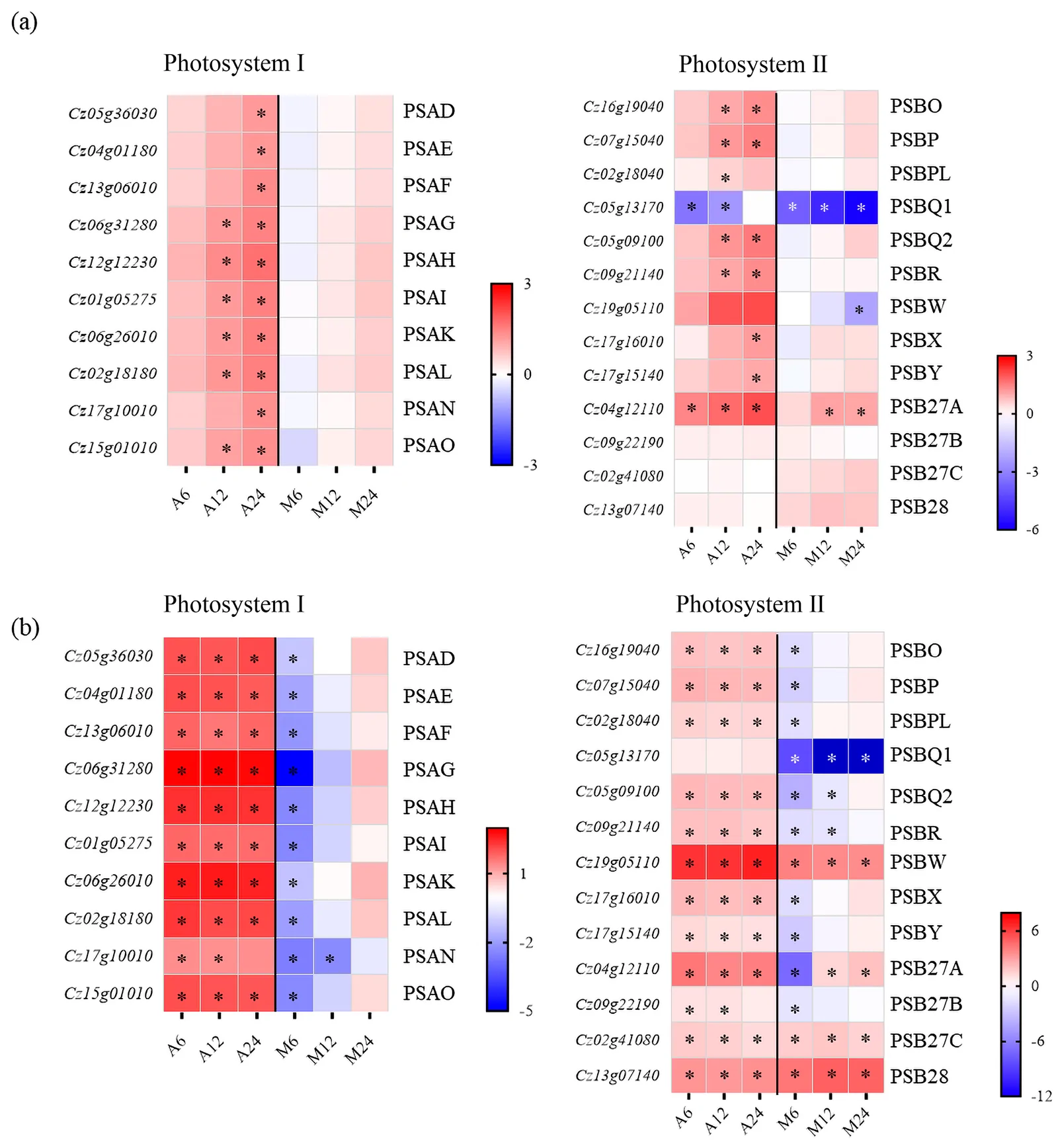
Time-course expression of photosystem-related genes in the (**a**) *Cz-pkg* mutant and (**b**) *Cz*-WT wild type under 100 mM NaCl treatment. A and M denote autotrophic and mixotrophic culture conditions, respectively, and 6, 12, and 24 indicate the hours after NaCl treatment, with 0 h as the corresponding control. Colors represent log_2_ fold changes in transcript abundance relative to the corresponding 0 h control; red indicates upregulation, blue indicates downregulation, and white indicates little or no change. The numerical range of the color scale differs among heatmaps and should be interpreted according to the corresponding color bar. Asterisks (*) indicate genes showing significant differential expression relative to the corresponding 0 h control according to the DEG criteria described in Section 3.11. The long vertical black line separates autotrophic and mixotrophic conditions.

**Figure 4:**
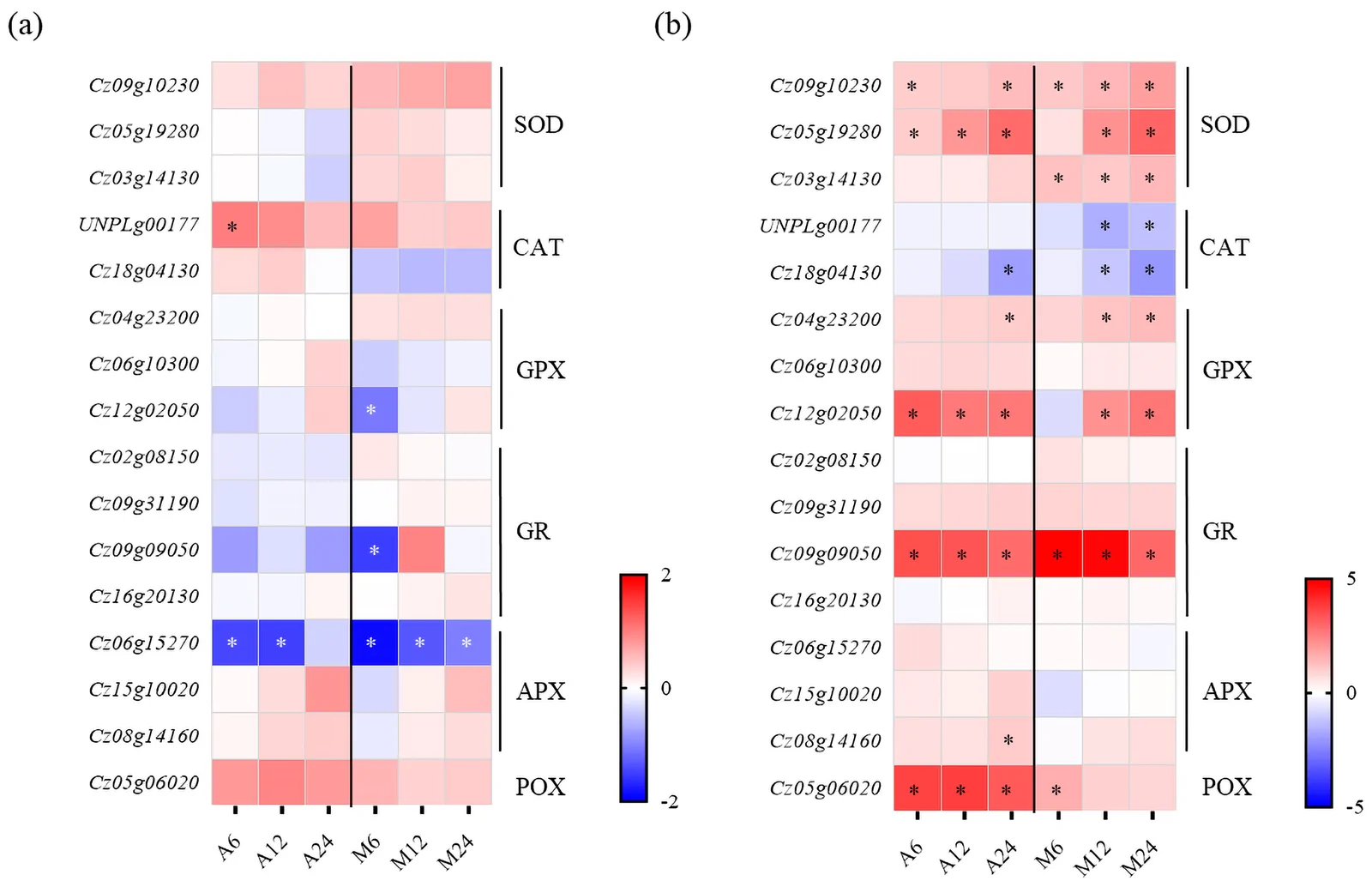
Time-course expression of ROS-scavenging genes in the (**a**) *Cz-pkg* mutant and (**b**) *Cz*-WT wild type under 100 mM NaCl treatment. A and M denote autotrophic and mixotrophic culture conditions, respectively. The numbers 6, 12, and 24 indicate the hours after NaCl treatment, with 0 h as the control. Asterisks (*) indicate genes showing significant differential expression relative to the corresponding 0 h control according to the DEG criteria described in Section 3.11. The long vertical black line separates autotrophic and mixotrophic conditions.

**Figure 5:**
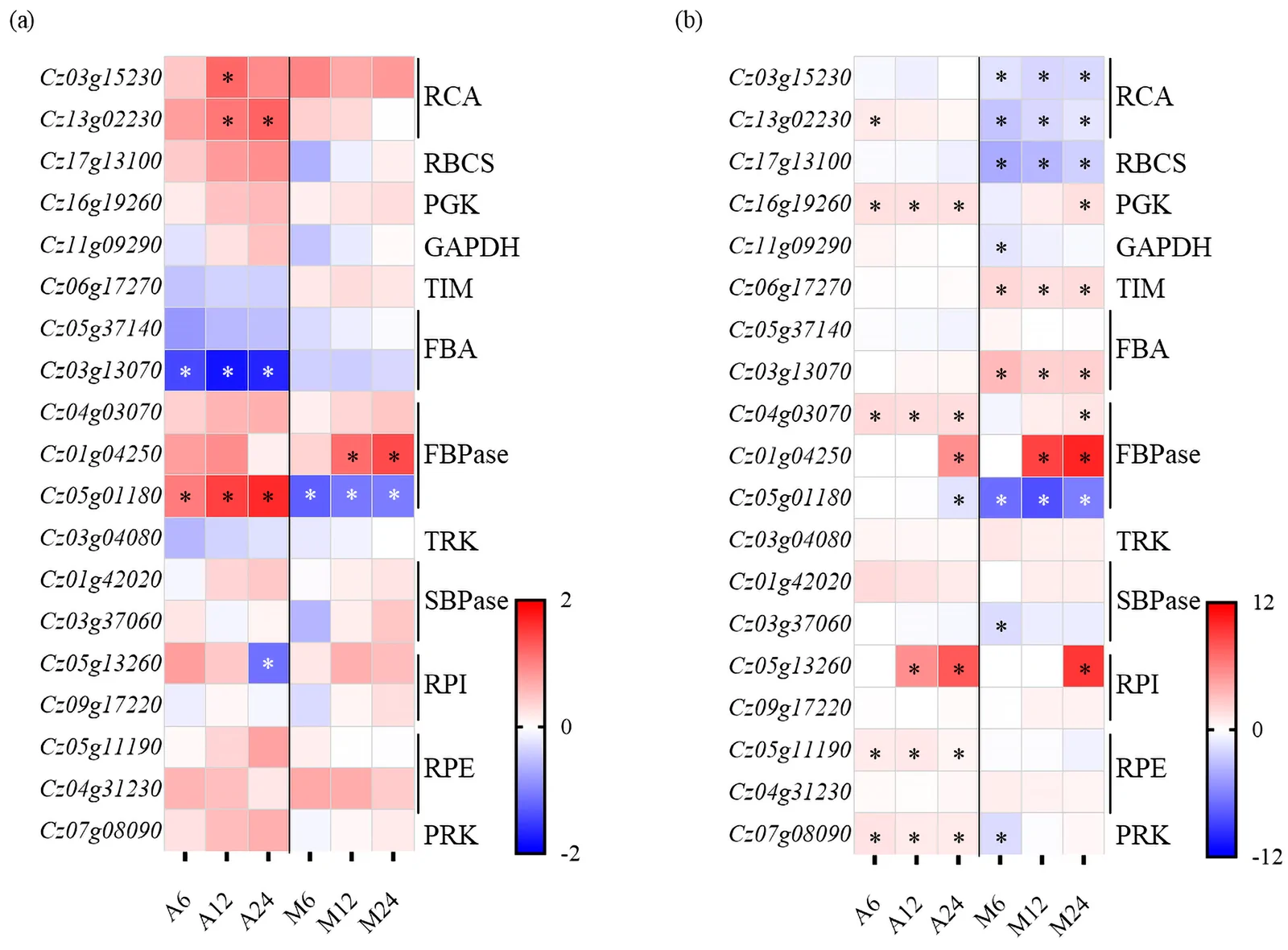
Time-course expression of Calvin cycle-related genes in the (**a**) *Cz-pkg* mutant and (**b**) *Cz*-WT wild type under 100 mM NaCl treatment. A and M denote autotrophic and mixotrophic culture conditions, respectively. The numbers 6, 12, and 24 indicate the hours after NaCl treatment, with 0 h as the control. Asterisks (*) indicate genes showing significant differential expression relative to the corresponding 0 h control according to the DEG criteria described in Section 3.11. The long vertical black line separates autotrophic and mixotrophic conditions.

**Figure 6:**
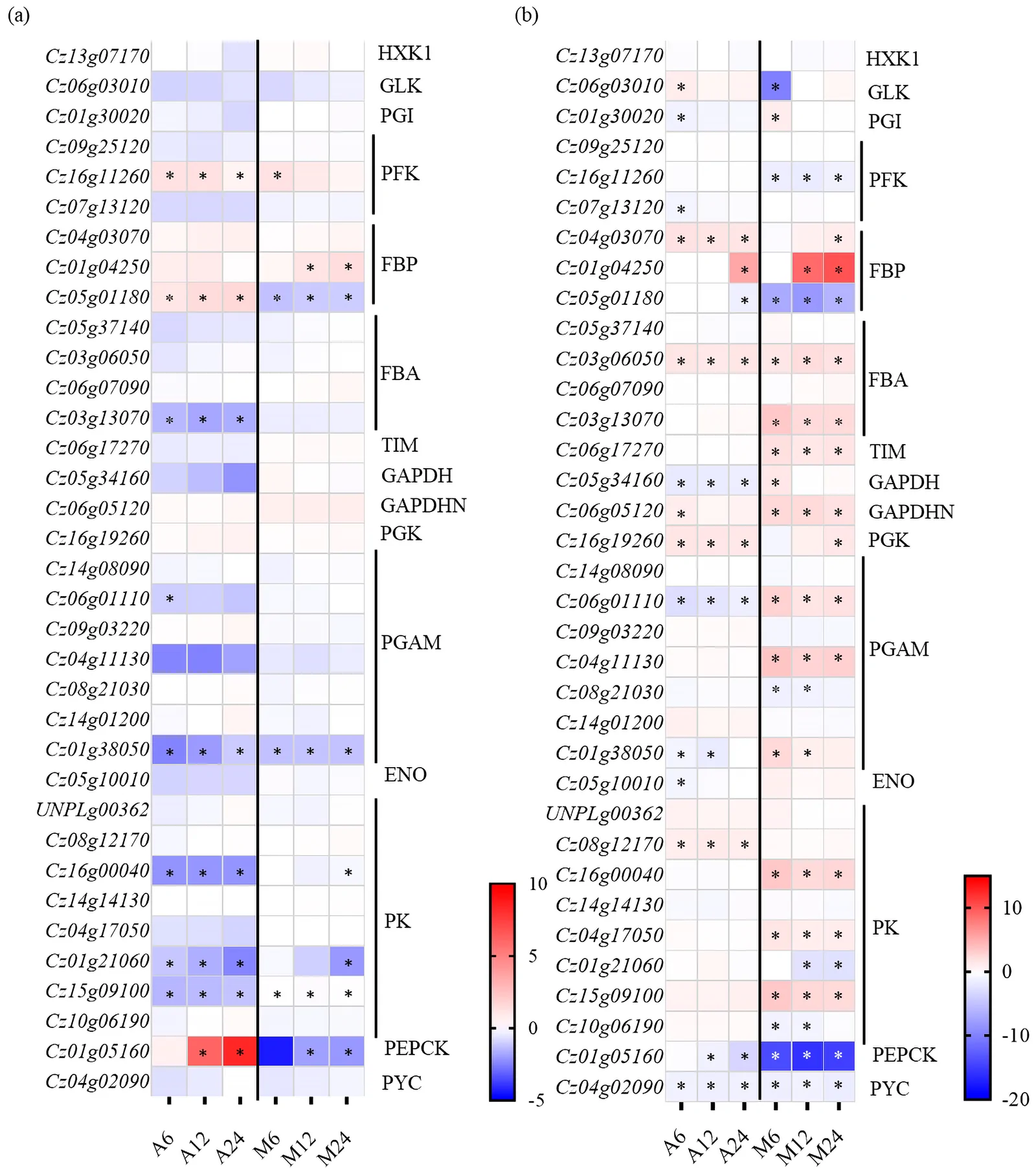
Time-course expression of genes related to EMP pathway and gluconeogenesis in the (**a**) *Cz-pkg* mutant and (**b**) *Cz*-WT wild type under 100 mM NaCl treatment. A and M denote autotrophic and mixotrophic culture conditions, respectively. The numbers 6, 12, and 24 indicate the hours after NaCl treatment, with 0 h as the control. Asterisks (*) indicate genes showing significant differential expression relative to the corresponding 0 h control according to the DEG criteria described in Section 3.11. The long vertical black line separates autotrophic and mixotrophic conditions.

**Figure 7:**
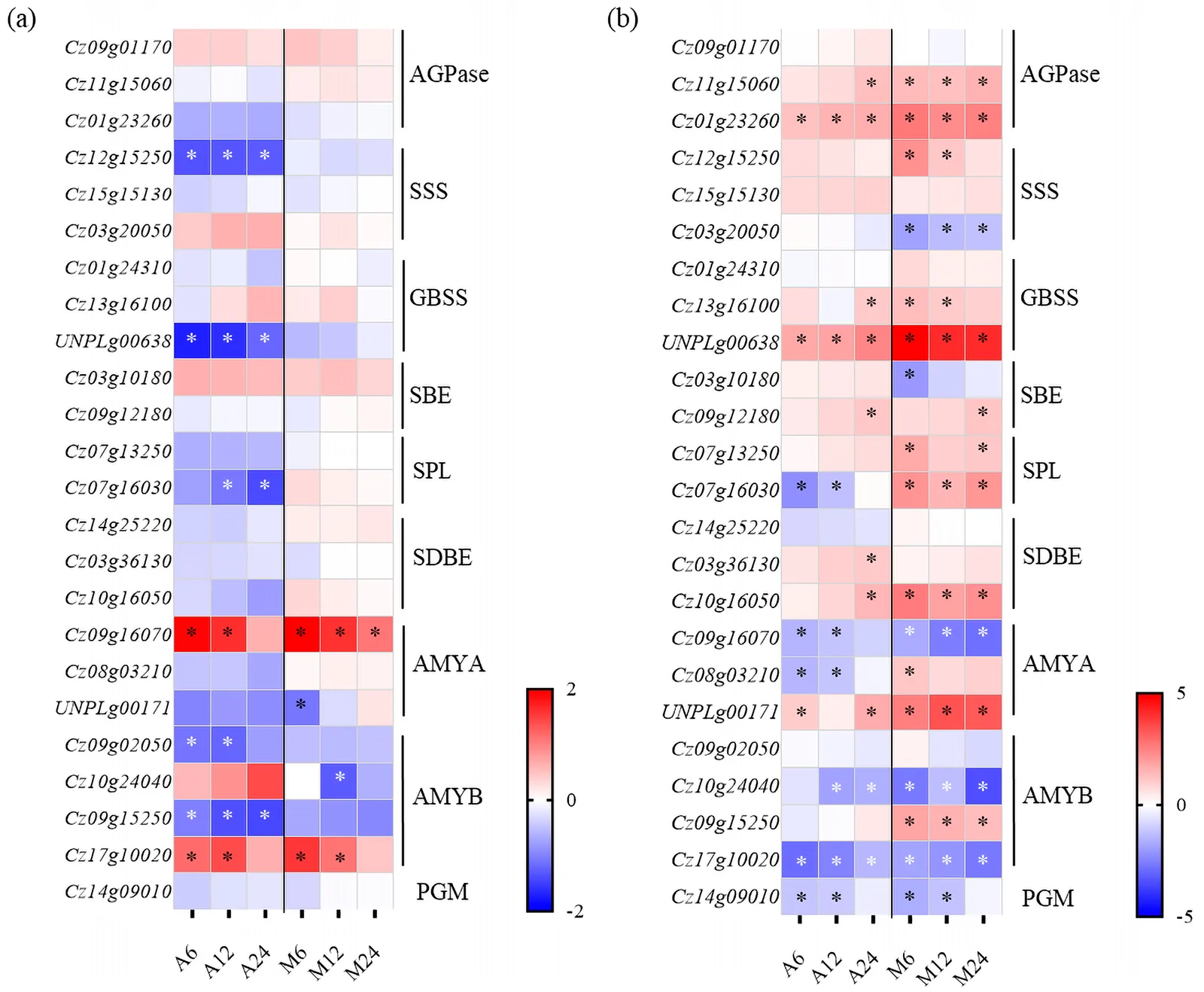
Time-course expression of starch metabolism-related genes in the (**a**) *Cz-pkg* mutant and (**b**) *Cz*-WT wild type under 100 mM NaCl treatment. A and M denote autotrophic and mixotrophic culture conditions, respectively. The numbers 6, 12, and 24 indicate the hours after NaCl treatment, with 0 h as the control. Asterisks (*) indicate genes showing significant differential expression relative to the corresponding 0 h control according to the DEG criteria described in Section 3.11. The long vertical black line separates autotrophic and mixotrophic conditions.

**Figure 8:**
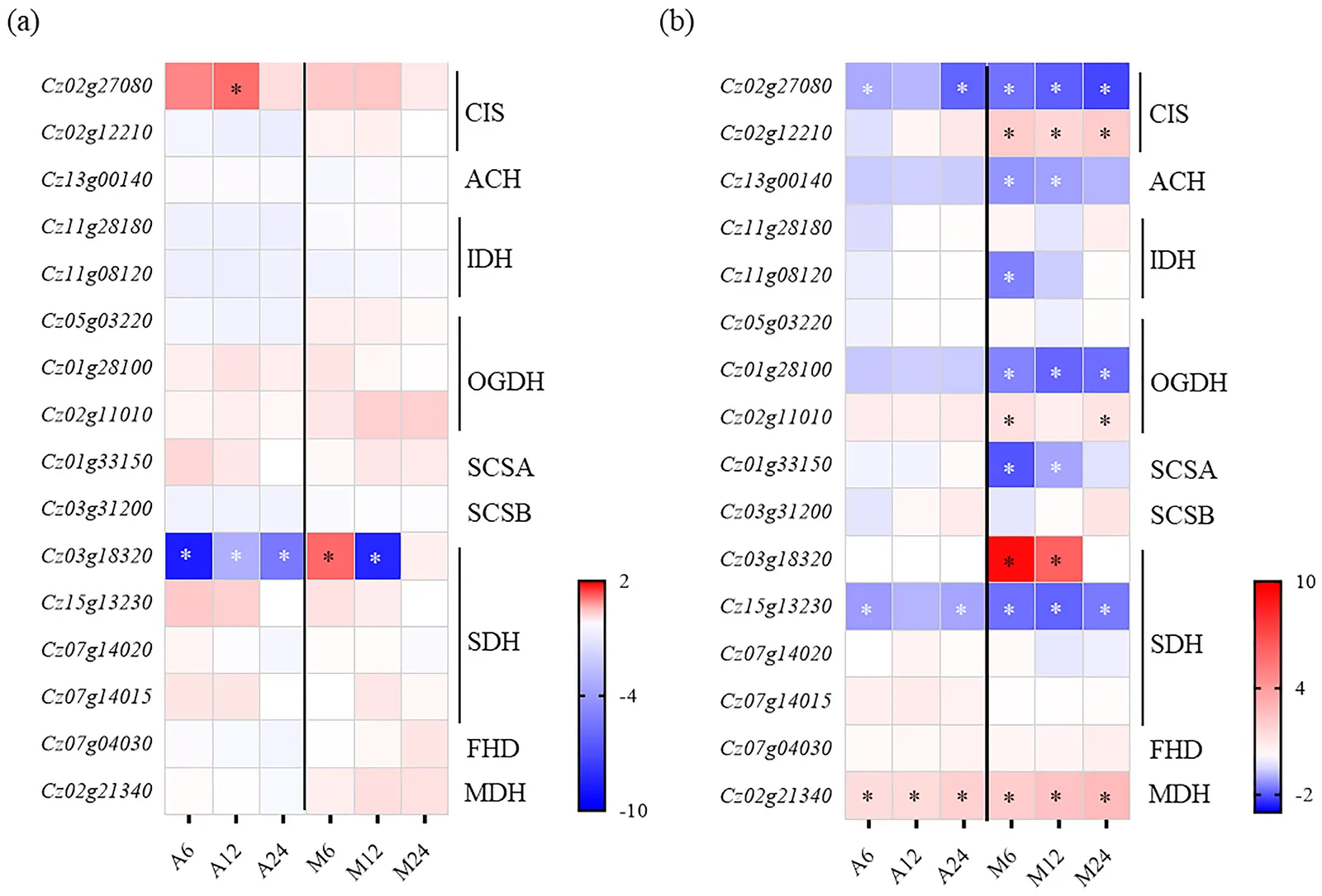
Time-course expression of TCA cycle-related genes in the (**a**) *Cz-pkg* mutant and (**b**) *Cz*-WT wild type under 100 mM NaCl treatment. A and M denote autotrophic and mixotrophic culture conditions, respectively. The numbers 6, 12, and 24 indicate the hours after NaCl treatment, with 0 h as the control. Asterisks (*) indicate genes showing significant differential expression relative to the corresponding 0 h control according to the DEG criteria described in Section 3.11. The long vertical black line separates autotrophic and mixotrophic conditions.

**Table 1:** Acid-hydrolysable amino acid composition of *Cz*-WT and *Cz-pkg* treated with 100 mM NaCl.

Amino Acid Composition	*Cz*-WT (mg/g DW)	*Cz-pkg* (mg/g DW)
Gly	24.29 ± 0.03	18.20 ± 0.41
Ala	40.27 ± 0.63	33.19 ± 1.04
Ser	17.34 ± 0.36	13.59 ± 0.21
Pro	20.35 ± 0.66	15.96 ± 0.46
Val	30.83 ± 0.07	24.63 ± 0.86
Thr	26.64 ± 0.61	19.73 ± 0.98
Cys	1.95 ± 0.10	1.37 ± 0.10
Ile	17.19 ± 0.34	13.22 ± 0.34
Asp	0.07 ± 0.01	0.06 ± 0.03
Glu	43.75 ± 1.13	29.39 ± 2.25
His	58.73 ± 0.51	42.10 ± 1.70
Phe	4.32 ± 1.40	3.50 ± 1.37
Arg	8.95 ± 0.42	6.25 ± 0.21
Trp	17.41 ± 0.50	14.02 ± 0.24
Lys	100.33 ± 1.73	21.74 ± 0.99
Tyr	N.D.	N.D.
Leu	33.98 ± 0.61	23.48 ± 0.45

Notes: These measurements represent the acid-hydrolysable amino acid composition of biomass. Values are expressed as mg/g DW (dry weight) and represent amino acids recovered after acid hydrolysis of freeze-dried biomass. Under the hydrolysis conditions used, Asn and Gln are converted to Asp and Glu, respectively, and therefore were not quantified separately. Tyr was not detected under the analytical conditions used. This may reflect low Tyr recovery during sample hydrolysis and analysis rather than its complete absence from the biomass, as Tyr recovery can be affected by acid hydrolysis conditions.

**Table 2:** Nucleotide composition of DNA and RNA in *C. zofingiensis*.

Nucleotide	DNA (%)	RNA (%)
A	24.5	24.5
T (U)	24.5	24.5
G	25.5	25.5
C	25.5	25.5

**Table 3:** Normalized ^13^C-MFA flux estimates of central carbon metabolism in *Cz*-WT and *Cz-pkg* under 100 mM NaCl treatment.

Reaction	Reaction Formula	Normalized Flux	
		*Cz-pkg*	*Cz*-WT
r_1_	G6P → F6P	82.3	82.0
r_2_	6PG + NADP → Ru5P + CO_2_ + NADPH	36.3	36.2
r_3_	Ru5P + CO_2_ → F6P + NADPH	36.3	36.2
r_4_	F6P + ATP → FBP + ADP	82.3	82.0
r_5_	FBP ⇌ DHAP + GAP	82.3	82.0
r_6_	DHAP → GAP	82.3	82.0
r_7_	GAP → DHAP	0	0
r_8_	GAP → PEP + ATP + NADPH	172.3	174.3
r_9_	PEP → PYR + ATP	105.6	126.2
r_10_	PYR → AcCoA + NADH + CO_2_	70.5	106.5
r_11_	PYR + ATP + CO_2_ → OAA	/	/
r_12_	PEP + CO_2_ → OAA + ATP	15.6	180.3
r_13_	OAA + ATP → PEP + CO_2_	81.1	185.8
r_14_	AcCoA + OAA → ICT	45.9	68.7
r_15_	ICT → AKG + NADH + CO_2_	45.9	68.7
r_16_	AKG → SUC + NADH + GTP + CO_2_	42.3	67.0
r_17_	SUC → MAL + FADH_2_	42.3	67.0
r_18_	MAL → OAA + NADH	44.7	94.6
r_19_	AcCoA → AcCoA (Cyt)	/	/
r_20_	AcCoA (Cyt) + ATP + CO_2_ → MalCoA	/	/
r_21_	MalCoA → MalACP	/	/
r_22_	AcCoA (Cyt) + ACP → AcACP	/	/
r_A_	Glc + ATP → G6P + ADP	100	100

Notes: Fluxes were estimated from the averaged isotopomer data of three independent biological replicates and normalized by setting the glucose-to-G6P flux to 100 (rA). Values represent normalized flux point estimates rather than absolute metabolic rates. Glc, glucose; G6P, glucose 6-phosphate; F6P, fructose 6-phosphate; 6PG, 6-phosphogluconate; Ru5P, ribulose 5-phosphate; FBP, fructose 1,6-bisphosphate; DHAP, dihydroxyacetone phosphate; GAP, glyceraldehyde 3-phosphate; PEP, phosphoenolpyruvate; PYR, pyruvate; AcCoA, acetyl-CoA; OAA, oxaloacetate; ICT, isocitrate; AKG, α-ketoglutarate; SUC, succinate; MAL, malate; MalCoA, malonyl-CoA; MalACP, malonyl-ACP; AcACP, acetyl-ACP; ACP, acyl carrier protein; Cyt, cytosolic; NADP/NADPH, oxidized/reduced nicotinamide adenine dinucleotide phosphate; NADH, reduced nicotinamide adenine dinucleotide; FADH_2_, reduced flavin adenine dinucleotide.

**Table 4:** Selected metabolite responses of *Cz-pkg* and *Cz*-WT to 100 mM NaCl treatment under autotrophic and mixotrophic conditions.

Metabolic Category	Metabolite	Autotrophic 12 h		Autotrophic 48 h		Mixotrophic 12 h		Mixotrophic 48 h	
		*Cz-pkg*	*Cz*-WT	*Cz-pkg*	*Cz*-WT	*Cz-pkg*	*Cz*-WT	*Cz-pkg*	*Cz*-WT
Carbohydrate metabolism	D-Glucose 6-phosphate	0.60 ± 0.06	0.68 ± 0.01	0.59 ± 0.06	0.56 ± 0.01	0.63 ± 0.02	0.57 ± 0.04	0.66 ± 0.08	0.57 ± 0.01
Pentose phosphate pathway	6-Phosphogluconic acid	0.85 ± 0.01	0.86 ± 0.02	0.85 ± 0.01	0.87 ± 0.01	0.86 ± 0.02	0.87 ± 0.01	0.82 ± 0.05	0.89 ± 0.01
TCA cycle	Citric acid	1.18 ± 0.02	0.94 ± 0.14	1.04 ± 0.09	1.06 ± 0.04	1.16 ± 0.05	1.19 ± 0.10	1.20 ± 0.07	1.33 ± 0.07
Amino-acid metabolism	Argininosuccinic acid	0.38 ± 0.05	0.72 ± 0.02	0.78 ± 0.03	0.73 ± 0.03	0.57 ± 0.18	0.75 ± 0.01	0.68 ± 0.17	0.81 ± 0.01
Amino-acid metabolism	N-Acetyl-L-glutamic acid	1.02 ± 0.01	0.84 ± 0.05	0.90 ± 0.07	1.09 ± 0.01	1.05 ± 0.02	1.40 ± 0.03	1.20 ± 0.09	1.55 ± 0.03
Amino-acid metabolism	Citrulline	0.82 ± 0.01	1.04 ± 0.04	0.82 ± 0.02	1.00 ± 0.04	1.00 ± 0.05	1.71 ± 0.03	1.22 ± 0.24	1.74 ± 0.06
Nucleotide metabolism	dTMP	0.80 ± 0.01	0.78 ± 0.03	0.69 ± 0.07	0.80 ± 0.01	0.65 ± 0.03	0.81 ± 0.04	1.19 ± 0.22	1.35 ± 0.01
Energy/redox metabolism	NADH	0.85 ± 0.02	0.64 ± 0.02	0.85 ± 0.04	0.70 ± 0.04	0.72 ± 0.04	0.63 ± 0.01	0.87 ± 0.11	0.89 ± 0.01

Notes: Values represent relative metabolite response ratios following 100 mM NaCl treatment. Red and blue shading indicate significant increases and decreases, respectively, relative to the corresponding untreated control (*p* < 0.05). Unshaded cells indicate no significant change. Complete metabolite datasets are provided in Tables S3 and S4.

## Data Availability

All relevant data supporting the findings of this study are contained within the article and its Supplementary Materials. Raw data (RNA seq, physiological and biochemical) are available from the authors upon reasonable request.

## Supplementary Materials

The following supporting information can be downloaded at https://www.mdpi.com/article/10.3390/md24100337/s1, Table S1: Fatty acid composition (mass% of total fatty acids) of *Cz*-WT under mixotrophic conditions at different NaCl concentrations; Table S2: Fatty acid composition (mass% of total fatty acids) of *Cz-pkg* under mixotrophic at different NaCl concentrations; Table S3: Changes in metabolite content of *Cz-pkg* under 100 mM NaCl treatment; Table S4: Changes in metabolite content of *Cz*-WT under 100 mM NaCl treatment; Table S5: HPLC gradient program for amino acid analysis.

## Abbreviations

The following abbreviations are used in this manuscript:

^13^C-MFA	^13^C metabolic flux analysis
TCA	tricarboxylic acid
PPP	pentose phosphate pathway
PEP	phosphoenolpyruvate
PYR	pyruvate
OAA	oxaloacetate
AcCoA	acetyl-CoA
DCW	dry cell weight
LC-MS	liquid chromatography–mass spectrometry
DEG	differentially expressed gene
PGPS	phosphatidylglycerophosphate synthase

## Appendix A

### The Metabolic Pathways of the Network Described in Section 3.10

- Glycolysis (Embden–Meyerhof–Parnas pathway)
  1. Gluc + ATP → G6P + ADP
  2. F6P + ATP → FBP + ADP
  3. FBP ⇌ DHAP + GAP
  4. DHAP ⇌ GAP
  5. GAP + NAD + ADP + Pi ⇌ 3PG + ATP + NADH
  6. 3PG ⇌ PEP
  7. PEP + ADP → PYR + ATP
- Starch Biosynthesis
  8. G6P + ATP → ADP + ADPG
- PPP Pathway
  9. G6P + NADP → 6PG + NADPH
  10. 6PG + NADP → Ru5P + CO_2_ + NADPH
  11. Ru5P ⇌ X5P
  12. Ru5P ⇌ R5P
  13. X5P + R5P ⇌ GAP + S7P
  14. X5P + E4P ⇌ GAP + F6P
  15. S7P + GAP ⇌ E4P + F6P
- TCA Cycle
  16. PYR + NAD → AcCoA + CO_2_ + NADH
  17. AcCoA + OAA → CIT
  18. CIT ⇌ ICIT
  19. ICIT + NADP ⇌ AKG + CO_2_ + NADPH
  20. AKG + NAD → SucCoA + CO_2_ + NADH
  21. SucCoA + ADP + Pi ⇌ SUC + ATP
  22. SUC + FAD ⇌ FUM + FADH_2_
  23. FUM ⇌ MAL
  24. MAL + NAD ⇌ OAA + NADH
- Glyoxylate Cycle
  25. ICIT → Glyox + SUC
  26. AcCoA + Glyox → MAL
- Amphibolic Pathway
  27. ATP + CIT → ADP + OAA + AcCoA
  28. MAL + NADP → PYR + CO_2_ + NADPH
  29. PEP + CO_2_ → OAA + Pi
  30. OAA + ATP → PEP + CO_2_ + ADP
- Fatty Acid Biosynthesis
  31. DHAP + NADH ⇌ Glyc3P + NAD
  32. 8AcCoA + 7ATP + 14NADPH → C16:0 + 7ADP + 14NADP
  33. C16:0 + NADH + O_2_ → C16:1 + NAD
  34. 9AcCoA + 8ATP + 16NADPH → C16:0 + 8ADP + 16NADP
  35. C18:0 + NADH + O_2_ → C18:1 + NAD
  36. C18:1 + NADH + O_2_ → C18:2 + NAD
  37. C18:2 + NADH + O_2_ → C18:3 + NAD
- Amino Acid Biosynthesis
  38. AKG + NADPH + NH_3_ → Glu + NADP
  39. Glu + ATP + NH_3_ → Gln + ADP + Pi
  40. Glu + 2NADPH + ATP → Pro + 2NADP + ADP + Pi
  41. Glu + CO_2_ + Gln + NADPH + Asp + AcCoA + 5ATP → Arg + AKG + NADP + FUM + Ac + 5ADP + 5Pi
  42. OAA + Glu → Asp + AKG
  43. Asp + NH_3_ + 2ATP → Asn + 2ADP + 2Pi
  44. PYR + Glu → Ala + AKG
  45. 3PG + Glu + NAD → Ser + NADH + AKG + Pi
  46. Ser + THF ⇌ Gly + MEETHF
  47. Gly + THF + NAD ⇌ CO_2_ + MEETHF + NH_3_ + NADH
  48. Thr + NAD → Gly + AcCoA + NADH
  49. Ser + AcCoA + SO_4_ + 3ATP + 4NADPH → Cys + Ac + 4NADP + 3ADP + 3Pi
  50. Asp + PYR + Glu + 2NADPH + ATP + SucCoA → LL-DAP + AKG + 2NADP + ADP + Pi + Suc
  51. LL-DAP → Lys + CO_2_
  52. Asp + 2NADPH + 2ATP → Thr + 2NADP + 2ADP + 2Pi
  53. Asp + METHF + Cys + 2NADPH + ATP + SucCoA → Met + PYR + 2NADP + ADP + Pi + Suc + NH_3_ + THF
  54. 2PYR + NADPH + Glu → Val + CO_2_ + NADP + AKG
  55. 2PYR + AcCoA + Glu + NADPH + NAD → Leu + 2CO_2_ + AKG + NADP + NADH
  56. Thr + PYR + Glu + NADPH → Ile + CO_2_ + AKG + NADP + NH_3_
  57. E4P + 2PEP + Glu + NADPH + ATP → Phe + CO_2_ + AKG + NADP + ADP + 4Pi
  58. E4P + 2PEP + Glu + NADPH + NAD + ATP → Thr + CO_2_ + AKG + NADP + NADH + ADP + 4Pi
  59. E4P + 2PEP + R5P + Ser + Gln + NADPH + 3ATP → Trp + CO_2_ + Pyr + GAP + Glu + NADP + 3ADP + 6Pi
  60. R5P + FTHF + Gln + Asp + 5ATP + 2NAD → His + 2NADH + AKG + FUM + 5ADP + 6Pi + THF
- Oxidative Phosphorylation
  61. NADH + 0.5O_2_ + 3ADP + 3Pi → 3ATP + NAD
  62. FADH_2_ + 0.5O_2_ + 2ADP + 2Pi → 2ATP + FAD
- Transhydrogenation
  63. NADH + NADP ⇌ NADPH + NAD
  64. ATP hydrolysis
  65. ATP → ADP
- Transport Processes
  66. Gluc.Ext → Gluc
  67. CO_2_ → CO_2_.Ext
  68. O_2_.Ext → O_2_
- Nucleotide Biosynthesis
  69. 2ATP + Gln + CO_2_ → 2ADP + Glu + CarP
  70. R5P + ATP → PRPP + ADP
  71. 4ATP + CO_2_ + PRPP + Asp + Gly + 2Gln + 2FTHF → FUM + 2Glu + 4ADP + IMP + 2THF
  72. 3ATP + Gln + NAD + IMP → NADH + Glu + rGTP + 3ADP
  73. 3ATP + Asp + IMP → FUM + rATP
  74. CarP + Asp + NAD + PRPP + 2ATP → 2ADP + rUTP + CO_2_ + NADH
  75. ATP + Gln + rUTP → rCTP + ADP + Glu
  76. NADPH + rCTP → dCTP + NADP
  77. dCTP + METHF → dTTP + NH_3_ + THF
  78. NADPH + rATP → dATP + NADP
  79. NADPH + rGTP → dGTP + NADP
- MEP Pathway
  80. PYR + 3PG → DXP + CO_2_.
